# Variation around the dominant viral genome sequence contributes to viral load and outcome in patients with Ebola virus disease

**DOI:** 10.1186/s13059-020-02148-3

**Published:** 2020-09-07

**Authors:** Xiaofeng Dong, Jordana Munoz-Basagoiti, Natasha Y. Rickett, Georgios Pollakis, William A. Paxton, Stephan Günther, Romy Kerber, Lisa F. P. Ng, Michael J. Elmore, N’faly Magassouba, Miles W. Carroll, David A. Matthews, Julian A. Hiscox

**Affiliations:** 1 Institute for Infection, Veterinary and Ecological Sciences, University of Liverpool, Liverpool, UK; 2 NIHR Health Protection Research Unit in Emerging and Zoonotic Infections, Liverpool, UK; 3 Bernhard Nocht Institute for Tropical Medicine, Hamburg, Germany; 4 Singapore Immunology Network, A*STAR, Singapore, Singapore; 5 Public Health England, Salisbury, UK; 6 Laboratoire des fièvres hémorragiques en Guinée, Université Gamal Abdel Nasser de Conakry, Conakry, Guinea; 7 School of Cellular and Molecular Medicine, University of Bristol, Singapore, Singapore

**Keywords:** Ebola virus, Ebola virus disease, Virus genetics, Evolution, Patient outcome

## Abstract

**Background:**

Viral load is a major contributor to outcome in patients with Ebola virus disease (EVD), with high values leading to a fatal outcome. Evidence from the 2013–2016 Ebola virus (EBOV) outbreak indicated that different genotypes of the virus can have different phenotypes in patients. Additionally, due to the error-prone nature of viral RNA synthesis in an individual patient, the EBOV genome exists around a dominant viral genome sequence. The minor variants within a patient may contribute to the overall phenotype in terms of viral protein function. To investigate the effects of these minor variants, blood samples from patients with acute EVD were deeply sequenced.

**Results:**

We examine the minor variant frequency between patients with acute EVD who survived infection with those who died. Non-synonymous differences in viral proteins were identified that have implications for viral protein function. The greatest frequency of substitution was identified at three codon sites in the L gene—which encodes the viral RNA-dependent RNA polymerase (RdRp). Recapitulating this in an assay for virus replication, these substitutions result in aberrant viral RNA synthesis and correlate with patient outcome.

**Conclusions:**

Together, these findings support the notion that in patients who survived EVD, in some cases, the genetic variability of the virus resulted in deleterious mutations that affected viral protein function, leading to reduced viral load. Such mutations may also lead to persistent strains of the virus and be associated with recrudescent infections.

## Introduction

The major contribution to outcome in patients with Ebola virus disease (EVD) is viral load [1, 2]. Additionally, viral genome variants between patients may have different phenotype [3, 4]. Host factors [5–8] and co-infections [9] will also contribute to survival or a fatal outcome. Similar to other viruses with RNA genomes, the fidelity of EBOV RNA genome replication and RNA synthesis is influenced by the processivity of the L protein [10]. Additionally, there exist potential genome modifications resulting from nucleotide changes by the action of cellular proteins involved in RNA processing, including adenosine deaminases acting on RNA (ADARs) (inducing an A to G transition) [1, 11]. Any perturbation in the fidelity of genome replication that increases the rate of mutation can lead to error catastrophe where synonymous and non-synonymous changes lead to a degradation of viral protein function and a loss in viral RNA synthesis. This can be exploited therapeutically where drugs such as ribavirin [12] and favipiravir [13] can be used to drive the replication of RNA viruses towards being less fit.

The high nucleotide substitution rate can drive the selection of genotypic and phenotypic variants of EBOV that allows the virus to occupy new niches [1]. This is evidenced in the ability of EBOV to gain virulence in a small animal model to which it is not initially adapted to replicate (e.g. [14]). Concomitant adaptations in viral proteins, such as VP24, were present in the minor variant population before they became established as a dominant viral genome sequence [14]. Whilst variation and potential functional changes in the dominant viral genome sequence have been compared between patients, the understanding of minor variants and their frequency and how these contribute to the overall viral phenotype within a patient is unknown. The opportunity to investigate this in natural infections of EBOV in the human population will diminish with the potential use of medical countermeasures that may drive selection pressures such as favipiravir [15] and monoclonal antibody therapies [16] as supportive care becomes more prevalent.

To investigate the diversity underlying dominant viral genome sequences in patients with EBOV, we examined transcriptome data from blood samples that we had either previously sequenced [1, 5, 9] or that had been sequenced for this study. These samples represented diagnostic leftover material used by the European Mobile Laboratory (EMLab) and were sampled from patients with EVD at presentation to the Ebola Treatment Centre (ETC) in Guinea (2013–2016). The main differential was that the patients then went on to survive (termed hospitalised survivor) or die (termed hospitalised fatal). Our hypothesis was that viral populations may be different between individual patients in the acute phase EVD, and this contributed to the outcome.

## Results

To ensure equivalence of samples in terms of length of infection (which may have influenced viral load and genome diversity), self-reported information from patients was used. Samples were selected so that there was no significant difference in the mean-time to the onset of symptoms for the hospitalised survivor group (6.4 days) and for the hospitalised fatal group (5.9 days). In terms of sequence quality, data from the blood samples was included if the final assembled dominant EBOV genome sequence was longer than 18,800 nucleotides and without gap (N). Using both equivalence of infection and sequence read quality to filter for sample quality, the numbers of samples for comparison were 38 hospitalised survivors and 96 hospitalised fatal cases. The general statistics of these cases are summarised in Additional file 1: Table S1.

**Fig. 1:**
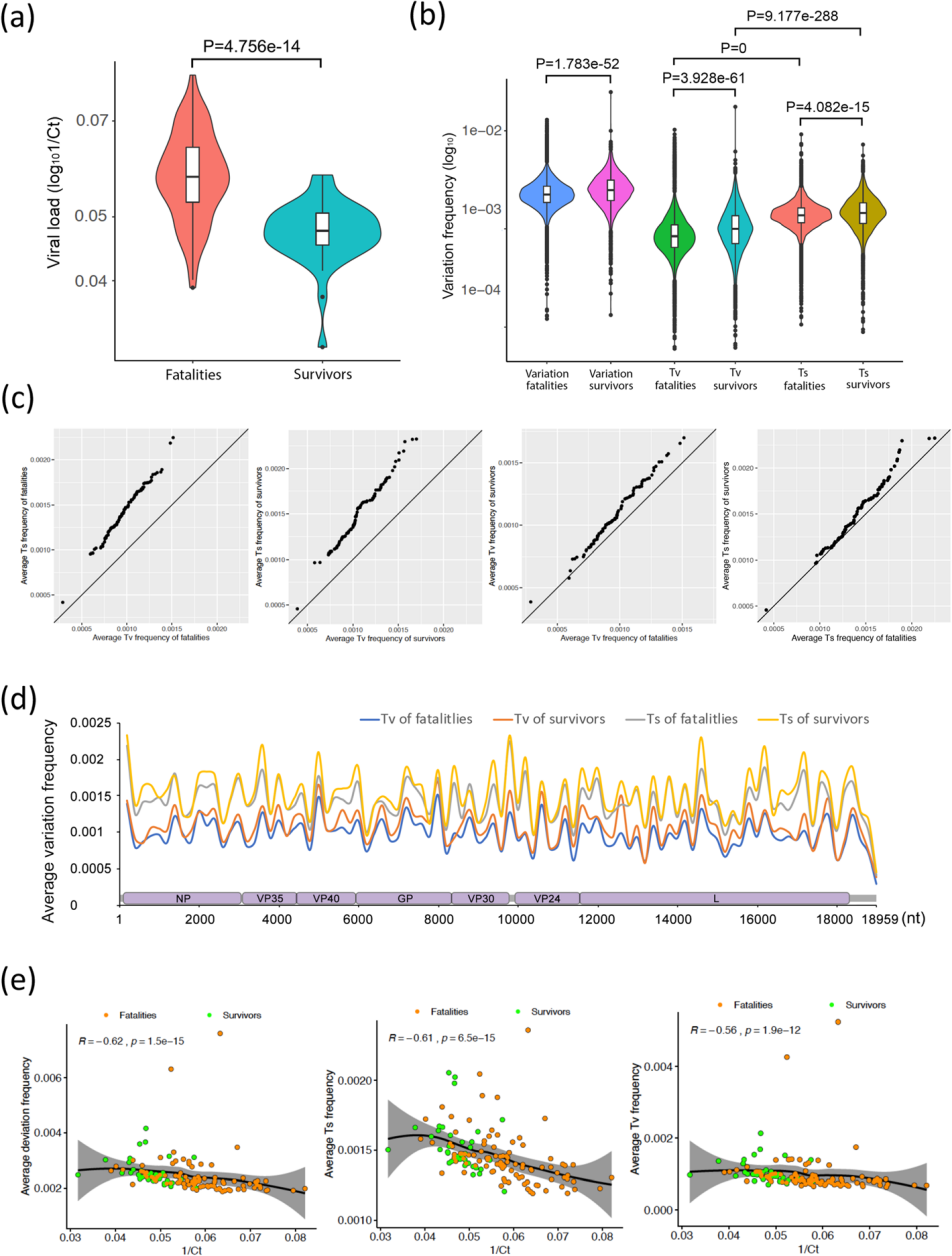
Ebola genome-wide mutational bias and viral load. **a** Comparison of the viral load (1/Ct) in hospitalised fatal versus hospitalised survived blood samples taken during the acute phase upon admission to an Ebola virus treatment centre. These data followed the normal distribution, so *P* values were calculated with a two-sided *t* test. **b** Comparison of the nucleotide variation from the dominant genome sequence in an individual patient in hospitalised fatal versus hospitalised survived cases. The variation frequency was calculated by transversion or transition deviations using a 200-nucleotide sliding window, and the *P* values were calculated with a one-sided Wilcoxon rank sum test as the data did not fit a normal distribution. **c** Q-Q plots were used to compare the distribution of the average nucleotide deviation in an individual patient in hospitalised fatal versus hospitalised survived cases using a 200-nucleotide sliding window along the genome for hospitalised survived versus hospitalised fatal cases. **d** Average nucleotide variation along the Ebola genome calculated by substitutions leading to either transversion or transition changes using a 200-nucleotide sliding window. **e** Comparison of the viral load (1/Ct) in hospitalised fatal versus hospitalised survived cases. A two-sided Spearman rank correlation test was used to estimate the correlation of the average nucleotide variation and viral load (1/Ct) of each sample from a patient, where the *R* value is the correlation coefficient ranging in − 1 (strong negative correlation) and + 1 (strong positive correlation), and *P* is the *P* value for this test

Reflecting the observations from Guinea [2], on average, the EBOV load was significantly higher in acute patients at presentation in the hospitalised fatal group compared to the hospitalised survivor group (Fig. 1a). To determine the viral genome population in an individual, reads were mapped to a reference EBOV genome to call a dominant viral genome sequence. This was used as a template in the second round of mapping to generate the reference EBOV genome for each individual patient. The variation in the four nucleotides at each site along the reference EBOV genome was counted for the individual patient. A sliding window of 200 nucleotides was used to derive and compare the average frequency in nucleotide variation along the genome.

The data provided information on the dominant viral genome sequence and the frequency and position of minor variants in the EBOV genome between the hospitalised survivor group and the hospitalised fatal group. These corresponded to the transition and transversion variations from the dominant viral genome sequence (Fig. 1b, c). In general, the frequency of minor variants which represented transitions was higher than the frequency of transversions. The variation in the frequency of both transition and transversion deviations was broader in the hospitalised survivor group compared to the hospitalised fatal group (Fig. 1b). To investigate the distribution of these deviations across the genome, the average frequency of the minor variants was plotted along the EBOV genome (Fig. 1d). This showed there were regions along the genome, including the L gene, that exhibited greater deviation from the dominant viral genome sequence and also that in a region of GP, transversions were more frequent than transitions in EBOV genomes from hospitalised survivors compared to hospitalised fatal cases (Additional file 1: Fig. S1).

The frequency of deviation from the dominant viral genome sequence may have been influenced by viral load, and the more genomes present, the more variation might exist. To investigate this, a Spearman rank correlation test was performed (Fig. 1e). This showed that the frequency of transition and transversion deviations from the dominant viral genome sequence was negatively related to viral load. The data implied a greater diversity in EBOV genomes in patients with lower viral loads compared to patients with high viral loads. A generalised linear model (glm) was used to investigate this observation and showed no significant difference in the slope and intercept between survivors and fatalities in any plot of Fig. 1e (Additional file 1: Table S2). This suggested, in dominant viral genome sequence present in hospitalised survivors and hospitalised fatal cases at presentation, with the same viral load, there were no differences in transitions or transversions.

The minor variant population may have resulted in non-synonymous changes that had functional divergence from the dominant viral genome sequence, resulting in differences in the primary amino acid sequence from that coded by dominant viral genome. This would lead to neutral, loss or gain in function of the affected viral protein. If the minor variant formed a significant proportion of the virus population, then any changes in viral protein function due to the minor variant may have had an overall effect on the activity of the viral protein in the infection.

**Fig. 2:**
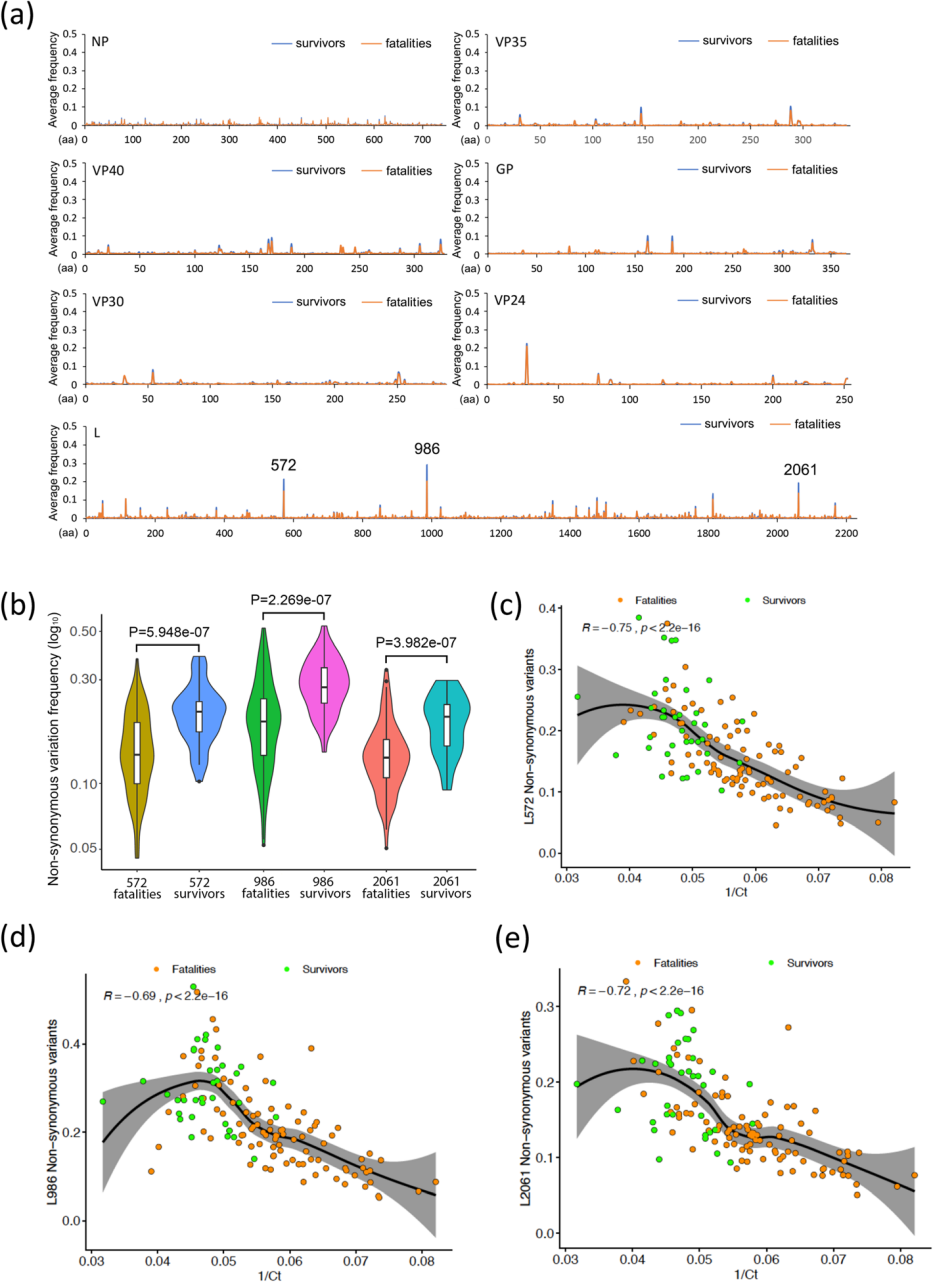
Analysis of non-synonymous changes and their correlation with viral load in acute patients. **a** The average non-synonymous variation in codon frequency at every amino acid site of each EBOV protein. **b** Comparison of the non-synonymous nucleotide substitution frequency in the L protein at positions 572, 986 and 2061, and the *P* values were calculated with a one-sided Wilcoxon rank sum test. **c** A two-sided Spearman rank correlation test was used to estimate the correlation of average non-synonymous deviation in viral genomes with viral load (1/Ct) at positions 572, 986 and 2061 in patients who were either hospitalised fatal versus hospitalised survived cases, where the *R* value is the correlation coefficient ranging in − 1 (strong negative correlation) and 1 (strong positive correlation), and *P* is *P* value for this test

**Fig. 3:**
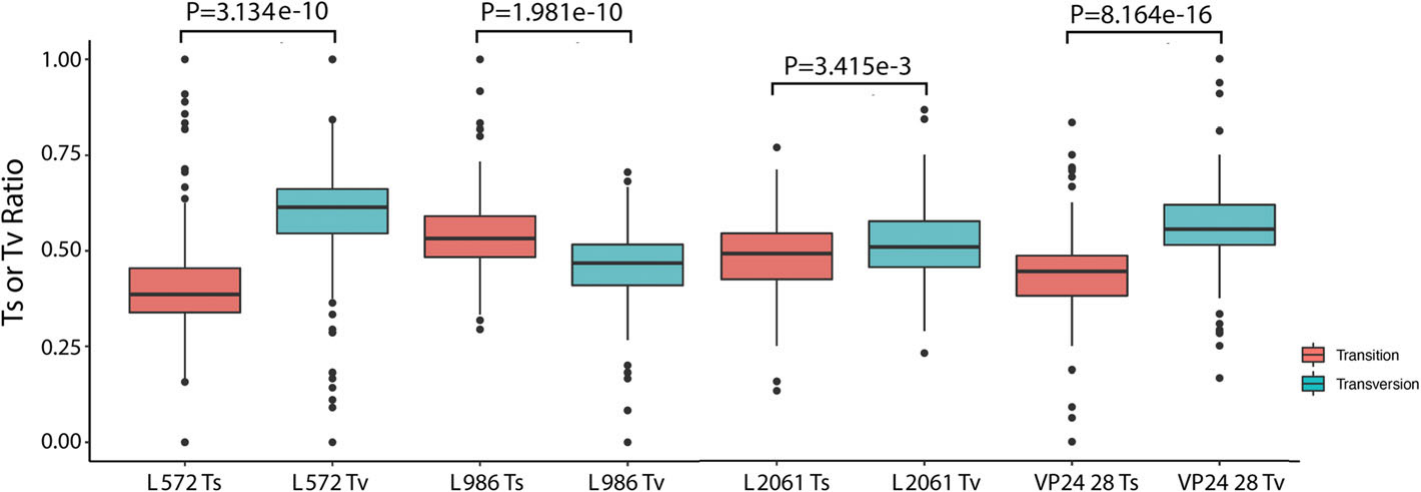
Comparison between Ts and Tv ratios that resulted in a non-synonymous change in positions 572, 986 and 2061 in the L protein and position 28 in VP24. The *P* values were calculated with a one-sided Wilcoxon rank sum test

To investigate this, the amino acid sequence space for all EBOV proteins was determined and the contribution of minor variants compared to the dominant viral genome sequence (Fig. 2a and Additional file 1: Fig. S2). In general, minor variants resulted in a small frequency of changes to the background protein sequence in all of the EBOV proteins (the dominant viral genome sequence by definition was still the majority sequence). However, in VP24 and L, there were several peaks where the frequency of the minor variants would have resulted in 20% or more of the protein space having a different amino acid at that position (Fig. 2a). The minor variant changes in VP24 led to one amino acid being present in the minor variants that was different from the dominant viral genome sequence but was similar for both hospitalised survivor and hospitalised fatal cases (Fig. 2a). In the L protein, three of these sites between hospitalised survivor and hospitalised fatal cases showed the highest difference with the most significant *P* values than all other amino acid sites of EBOV proteins: positions 572, 986 and 2061 (Fig. 2a, b; Additional file 1: Fig. S2). These deviations from the dominant viral genome sequence had a strong negative correlation with viral load (for each position the *R* value was less than − 0.69 (range − 1 to + 1) (Fig. 2c–e). These changes were mostly transversions, except at position 986, where these were predominately transitions (Fig. 3). Also, a glm showed significant differences of slope and intercept between survivors and fatalities in the data presented in Fig. 2c–e (Additional file 1: Table S2). This suggested that the frequency of non-synonymous changes at positions 572, 986 and 2061 of the L protein under the same viral load (1/Ct value) was significantly different between hospitalised survivors and hospitalised fatalities.

**Fig. 4:**
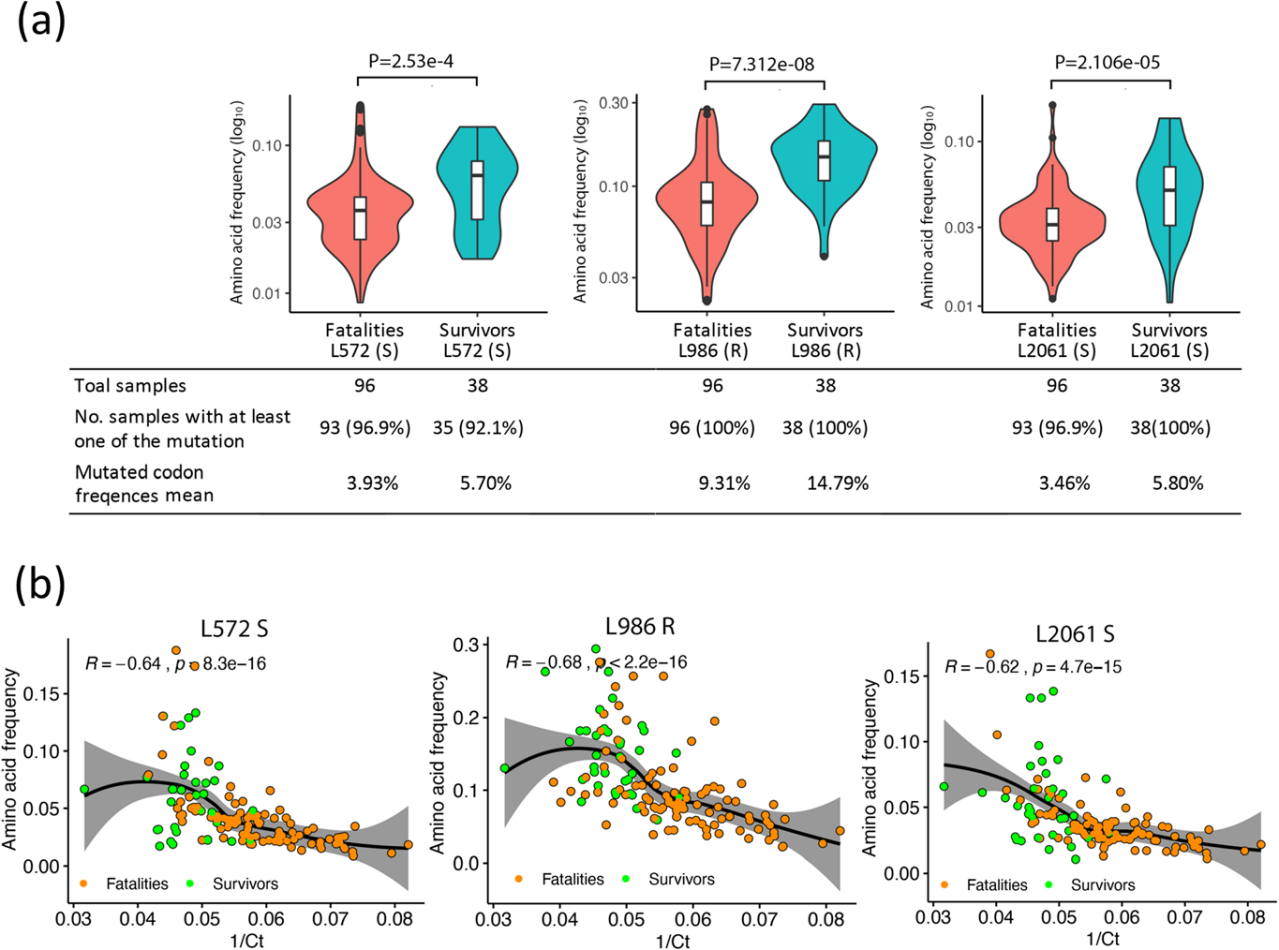
**a** Comparison of three amino acid variation frequencies in the L protein at positions 572, 986 and 2061. *P* values were calculated with the one-tailed Wilcoxon rank sum test. **b** A Spearman rank correlation test was used to estimate the correlation of these three amino acid variation frequencies with viral load (1/Ct) at positions 572, 986 and 2061, where the *R* value is the correlation coefficient ranging from − 1 (strong negative correlation) to + 1 (strong positive correlation), and *P* is the *P* value for this test. In **a** and **b**, only the samples with at least amino acid variation are shown

**Fig. 5:**
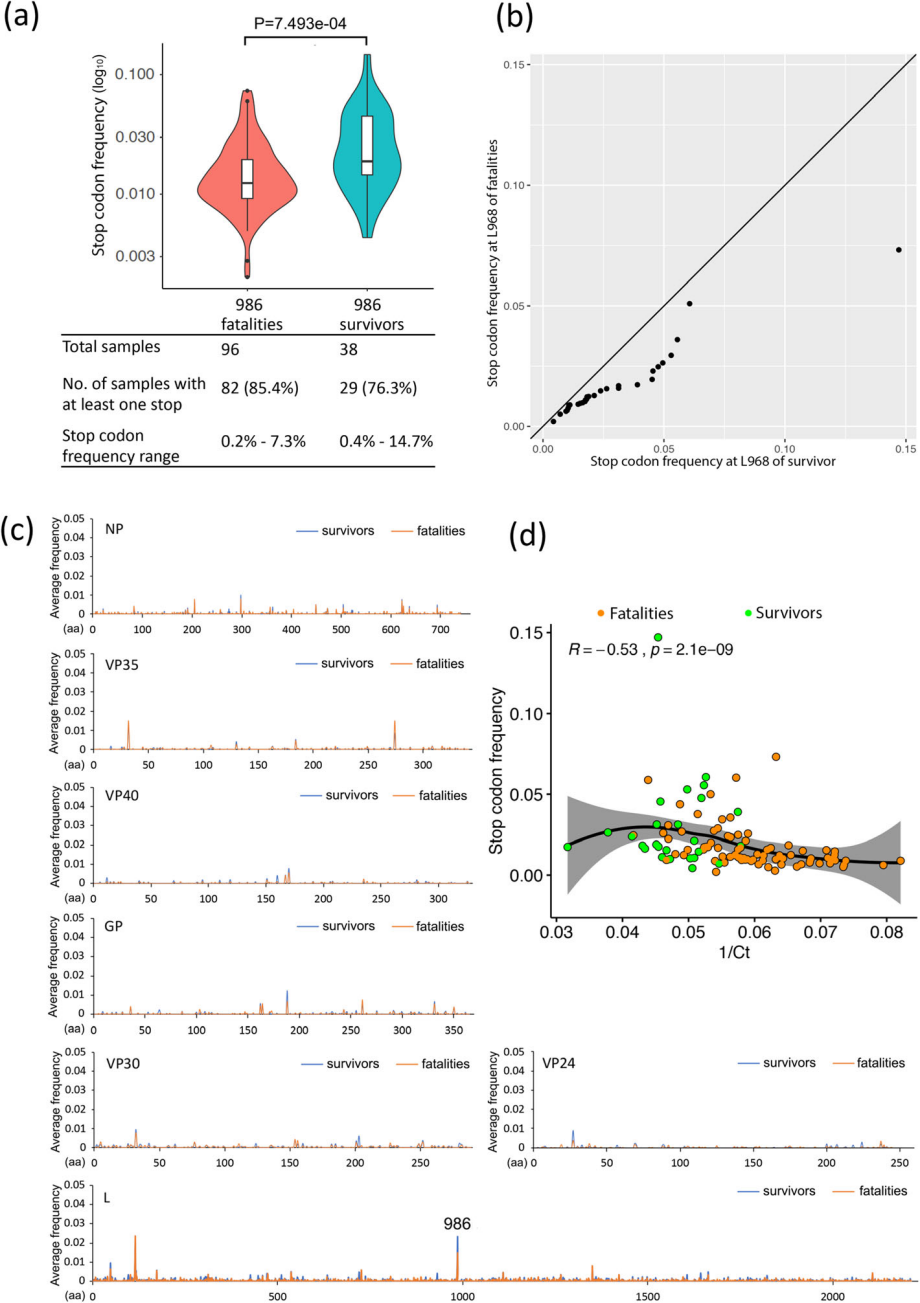
Analysis of the frequency of stop codon substitution in viral proteins and viral load. **a** Comparison of stop codon frequency in the L protein at position 986. *P* values were calculated with the one-sided Wilcoxon rank sum test, with the average stop codon frequency at position 986 in the EBOV L protein and compassion between hospitalised fatal and hospitalised survived cases. **b** A Q-Q plot was used to compare the distribution of the stop codon at position 986 in the L protein between hospitalised fatal and hospitalised survived cases. The values below the line suggest the data, i.e. the presence of the stop codon, was more frequent in the hospitalised survivor cases. **c** The summary of stop codon frequency in all EBOV proteins compared between hospitalised fatal and hospitalised survived cases. **d** A two-sided Spearman rank correlation test was used to estimate the correlation of stop codon frequency with viral load (1/Ct) at position 986, where the *R* value is the correlation coefficient ranging from − 1 (strong negative correlation) to + 1 (strong positive correlation), and *P* is the *P* value for this test. In **b**, **d** and **e**, only the samples with at least one stop codon are shown. In **a**, **c** and **d**, only the samples with at least one stop codon are shown

The predominant amino acid changes caused by minor variants at positions 572, 986 and 2061 in the L protein are shown in Additional file 1: Fig. S3. These include N572S, Q986R and F2061S and are more frequent in the EBOV minor variant population in the hospitalised survivor group than the hospitalised fatal group (Fig. 4a). Moreover, their usage correlated with a lower viral load in patients and therefore the hospitalised survivor category (Fig. 4b). One of the deviations from the dominant genome sequence in the L protein at position 986 was for a stop codon and would have resulted in a truncated L protein. This stop codon was present in a greater frequency, although in less patients, in the hospitalised survivor versus hospitalised fatal cases (Fig. 5a, b). For example, in one hospitalised survivor, the stop protein at position 986 reached a frequency of approximately 15% in the minor variant population. Indeed, stop codons were present at low frequency in all EBOV proteins (Fig. 5a), but it appeared more frequently at position 986 in the L protein than any other position in viral proteins (Fig. 5c). Similar to the other amino acid changes caused by minor variants at positions 572, 986 and 2061, the stop codon frequency at position 986 was more frequent in hospitalised survivors than hospitalised fatal cases (Fig. 5a, b) and was also negatively related to viral load (Fig. 5d). The glm showed significant differences in slope and intercept between data from hospitalised survivor and hospitalised fatal cases in Fig. 5d (Additional file 1: Table S2). This suggested that the stop codon was present in a greater frequency at position 986 of L protein under the same viral load (1/Ct value).

**Fig. 6:**
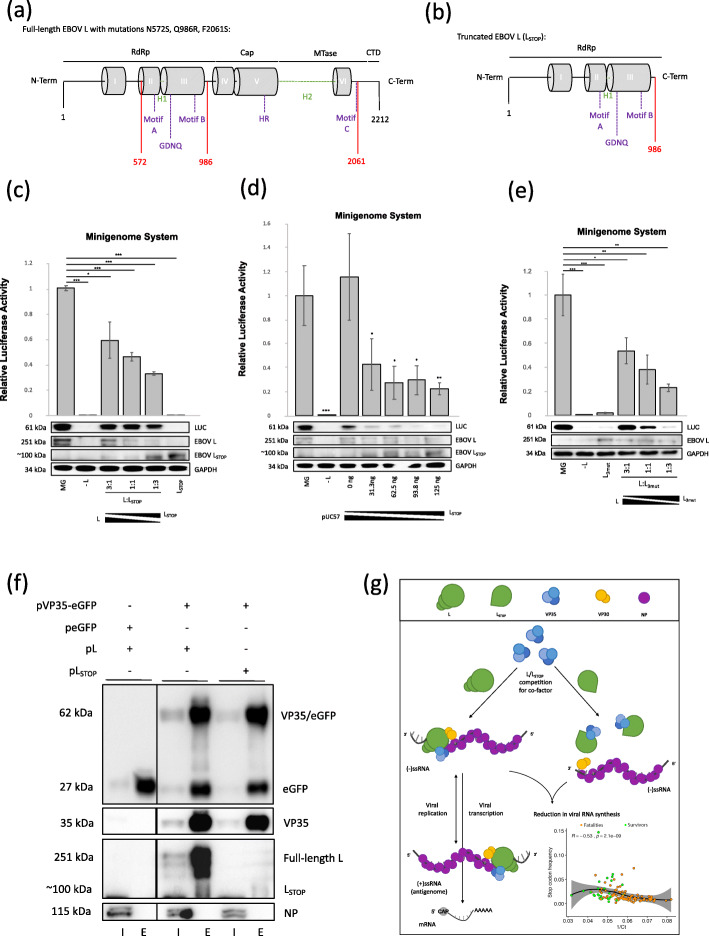
Functionality of L_STOP_ and L_3mut_ in an EBOV transcription/replication plasmid-based system (mini-genome) in cell culture. **a**, **b** Schematic diagrams of the conserved domains (grey boxes), functional motifs (purple) and variable regions or hinges (discontinuous green line) in EBOV L_3mut_ and L_STOP_. This diagram is based on data for filovirus and mononegavirus models for the L protein. Conserved blocks I–III constitute a RdRp, which is closely associated with a capping domain (Cap). Block VI has methyltransferase (MTase) activity, and downstream of this is located in a small C-terminal domain (CTD). Red highlighted the amino acid position where the **a** three most frequently found amino acid changes in the L protein at positions 572, 98s and 2061 are located and **b** the truncated protein due to the stop codon in L. **c** EBOV mini-genome system activity at different ratios between EBOV L and L_STOP_ and **d** at equal EBOV L but different L_STOP_ amounts. Results are shown as the mean ± S.D. from one experiment performed in triplicate. ***P < 0.001; **P < 0.01; *P < 0.05. Western blot for luciferase (LUC), EBOV L/L_STOP_ and house-keeping GAPDH protein abundance in cells transfected with the mini-genome system. **e** EBOV mini-genome system activity in the presence of the L, no L and L_3mut_ and at different ratios between L and L_3mut_. Blotting showed LUC and GAPDH abundance in cells transfected with the mini-genome system plasmids. **f** VP35-eGFP was used in a co-immunoprecipitation (coIP) assay to examine its interaction with EBOV L_STOP_. Blotting showed the presence of eGFP and viral proteins VP35/eGFP, VP35, L and NP in the cell lysates (input (I)) and coIP fraction (eluate (E)). **g** Proposed model of the competition between EBOV L and L_STOP_ for the viral RdRp co-factor VP35 and the potential reduction in the EBOV RNA synthesis observed in patients with lower viral load (inset panel)

We postulated that changes in the minor variant frequency and concomitant change in amino acid usage in the L protein at positions 572, 986 and 2061 would have had a negative impact on virus biology—given the correlation with reduced viral load in patients (Fig. 2c–e, Fig. 4b and Fig. 5d). The L protein of the filoviruses and the wider family of the *Mononegavirales* has a conserved structure with functional domains separated by hinge regions (Fig. 6a). Although the presence of a stop codon would produce a truncated protein (Fig. 6b), this may have remained biologically active as it was C-terminal of the catalytic domain for RNA synthesis [17]. Several studies have shown that individual domains of the L protein in *Mononegavirales* have biological activity, and exogenous sequence can be inserted into the hinge regions whilst still maintaining function [18–20]. Likewise, the expression of L protein within a specific range may be required for optimal viral RNA synthesis [21].

To investigate the activity of a truncated L protein due to the stop codon at position 986, and amino acid changes due to minor variants, we made use of a mini-genome system developed in the laboratory for Ebola virus Makona [22]. Here, viral proteins required for RNA synthesis, L, NP, VP30 and VP35, are provided *in trans* from helper expression plasmids. These drive the replication of the mini-genome and transcription of a luciferase reporter mRNA whose cDNA has been inserted between the 3′ and 5′ UTRs of the EBOV genome. Such systems have been shown to faithfully recapitulate viral RNA synthesis [23]. The insertion of the luciferase cDNA, and concomitant activity of the luciferase reporter protein, provides a rapid readout for functional analysis of viral proteins and variants, through substitution in the helper expression plasmids.

The activity of the truncated L protein, through the replacement of the Q at position 986 with a stop codon (referred to as L_STOP_), was compared to the wild-type L protein. Here, the activity of luciferase was compared between mini-genome systems supported by the L protein expression plasmid, or where this plasmid was excluded, or a combination of both the L protein and L_STOP_ expression plasmids or the L_STOP_ expression plasmid only. All of the other support plasmids, expressing NP, VP30 and VP35, were provided as normal (Fig. 6c, Additional file 1: Fig. S4). Western blot confirmed the expression of L and L_STOP_. In line with previous observations, excluding the L protein led to background observable luciferase activity compared to wild-type L protein (Fig. 6c, Additional file 1: Fig. S4). As the ratio of L_STOP_ to L expression plasmid was increased, there was a decreasing luciferase activity, such that with L_STOP_ only, the level of luciferase was not significantly different from excluding the L protein expression plasmid (Fig. 6c, Additional file 1: Fig. S4). This suggested that the L_STOP_ could not function as a RdRp. To investigate the potential loss of the overall function of the L protein activity, increasing amounts of the L_STOP_ expression plasmid were titrated in, with equivalent amounts of pUC57 added to maintain the total amount of DNA during transfection. Here, for all amounts of the L_STOP_ expression plasmid tested, there was a significant reduction in luciferase activity from using the L expression plasmid only (Fig. 6d, Additional file 1: Fig. S5). However, there was no significant difference in luciferase activity for all of the amounts of the L_STOP_. Given that N572S, Q986R and F2061S substitution as a result of minor variants could be present in the same patient (Additional file 1: Fig. S3). To evaluate the activity of these mutations in the context of the L protein, an expression variant called L_3mut_ was constructed where all three mutations were present. The data indicated that the activity of L_3mut_ was significantly reduced compared to the wild-type L protein (Fig. 6e, Additional file 1: Fig. S6), including when both wild-type L protein and L_3mut_ were expressed at the same time.

From this, we postulated that not only did these minor variant proteins have no (L_STOP_) or reduced activity (L_3mut_), they may also have acted as a sink to sequester other viral proteins required for viral RNA synthesis away from the dominant viral genome sequence for the L protein. To test this hypothesis, the capability of L_STOP_ to interact with VP35, a known polymerase cofactor for the L protein, and essential for replication and transcription [24–26], was examined. The ability of L_STOP_ to associate with VP35 was compared to L protein using a co-immunoprecipitation assay. Here, VP35 had been C-terminal tagged in frame with enhanced green fluorescence protein (eGFP) (forming VP35-eGFP) to allow co-immunoprecipitation with a highly specific single-chain antibody. This approach has been used to study the interacting partners of a wide variety of viral proteins, e.g. [22, 27, 28]. Although somewhat diminished compared to wild-type VP35, VP35-eGFP, in the context of the mini-genome system, still allowed the generation of luciferase activity (data not shown), suggesting the protein was still biologically active, through its interactions with the L protein. Co-immunoprecipitation indicated that VP35-eGFP could be used to pull down either the L protein or L_STOP_ protein but not NP (Fig. 6f, Additional file 1: Fig. S7). Overall, the data is supportive of a model (Fig. 6g) in which the presence of L_STOP_ protein contributes to the reduction in viral load in patients (Fig. 6g, inset panel) possibly through both the absence of RdRp activity and the ability to act as a sink for viral proteins otherwise required for RNA synthetic activity.

## Discussion

The analysis of minor variant populations of EBOV in infected patients paints an interesting picture for their contribution to the outcome of EVD. In this study, we focused on the diversity found in viral populations in a cohort of patients with EVD where a blood sample was taken during the acute phase, and where patients then went on to survive or die. RNA-seq was used to generate sequence data to derive the dominant viral genome sequence within a patient and establish the frequency of minor variants. Within this minor variant pool were potential non-synonymous changes that may have had profound effects on the activity of EBOV proteins. In the case of data from patients with differing outcomes associated with EVD, three amino acid substitutions that were present on the L protein in the minor variant viral population within a patient were shown to have phenotypic consequences for replication/transcription of the viral genome. Substitution of these amino acids in the dominant genome sequence for the L protein resulted in aberrant viral RNA synthesis in the context of a replicon system. We postulate these divergent proteins may also act as a sink for other viral (and host) proteins involved in RNA synthesis, which associate and function through protein to protein interactions. Therefore, the net effect resulted in reduced viral RNA synthesis, which may have contributed to the lower viral load observed in patients containing viral populations with these variants.

Viral genome variation is not the only contributor to the outcome, although it does play a significant role in the patients we analysed. How viral diversity may be generated inside a patient is unknown, and this may be related to an initial large viral population infecting a patient and/or host mechanisms. Infection may result from a single or a small number of founder viruses that emerge from a genetic bottleneck. This is supported by studies in a guinea pig model of infection, where specific variations in the genome sequence become predominant and are associated with gain of virulence [14]. These variations are present in the minor variant population and on the passage in the animal model become the dominant viral genome [29]. At a more general level, the correlation between viral genome diversity and severity of clinical symptoms has also been described for hepatitis C virus, where viral genome diversity in asymptomatic carriers was greater than in patients with severe disease and explained from a population genetics view [30].

Both host effects [5, 6, 31] and the presence of other infections [9] will undoubtedly contribute to the outcome in EVD. The host response to EBOV infection is a major determinant of disease severity in EVD [5], as well as the timing and magnitude of transcriptional regulatory networks, where early induction of inflammatory gene expression is associated with an asymptomatic course of the disease [32]. The host response at the acute stage in many of the patients covered in this study was investigated previously [5]. Here, read mapping to the human genome was used to identify and quantify changes in mRNA profiles between hospitalised survivors and hospital fatal infections. Digital cell quantification and flow cytometry was used to investigate differences in the immunological phenotype of the blood [5]. The host response may affect viral population genetics through selection pressure. The caveat is that our study and the work described [5] are based on a single snapshot of infection (on average day 6 post-infection based on self-reporting from a patient or their family) and therefore do not encompass the entire immune response, and in particular, the adaptative response. In patients who went on die, at the acute phase, there was a stronger upregulation of interferon signalling compared to patients who went on to survive. Also, in this latter group of patients, a greater abundance in NK cell populations was predicted [5]. A severe T cell dysfunction is associated with fatal EVD cases [31], and non-antigen-specific activation of lymphocytes T and B has been associated with a higher production of pro-inflammatory cytokines, which may lead to immune impairment [33, 34] and therefore influence the dominant viral genome and minor variant population. From a more innate/intrinsic immunity perspective, several proteins within the cell may affect viral genome sequences. The predominant transition variation in the EBOV genome was an A to G substitution (Additional file 1: Table S3) which is reminiscent of ADAR activity. Whether ADAR activity is more enhanced in hospitalised survivors versus hospitalised fatal cases is unknown. ADAR activity can be associated with cellular localisation and therefore was not possible to examine in the context of the diagnostic leftover RNA sample. However, we note that in EBOV-infected A549 cells, a human cell line with a strong innate response, ADAR transcripts were increased in abundance compared to mock-infected cells [35].

The potential attenuation of viral replication through variation in the L protein may have several implications. First, the data may provide mechanistic insight into why favipiravir would potentially be effective in human patients with lower viral loads [15]. In a non-human primate model of EVD, favipiravir has been shown to increase mutagenesis during EBOV RNA synthesis [13]. Therefore, together with the potentially higher variability of EBOV in patients who go on to survive (and have lower viral loads), flavipiravir may tip the balance towards error catastrophe during viral replication. Second, there have many cases of potential persistence associated with a recrudescence in EBOV infection, including in the testes [36], the ocular fluid [37] and the central nervous system [38]. These observations are also reflected in animal models of persistent asymptomatic infection with EBOV [39]. Recently, a model for the persistence of acute viral infections, such as those observed in paramyxoviruses, has been proposed [40]. EBOV has a similar genome organisation and replication strategy to paramyxoviruses. In this model, as the adaptive immune response develops during infection, genome variants are selected in which replication is suppressed [40]. This would lead to persistent infection at low levels, and the switch back to a lytic stage would be associated with amino acid reversions causing virulence [40]. In the paramyxovirus, parainfluenza virus type 5 (PIV), a single amino acid change in the viral P protein, a key component of the viral RNA polymerase complex, was found to control the switch from persistence to lytic replication [40]. The observation of the attenuating N572S, Q986R and F2061S variants in the EBOV L protein was associated with reduced polymerase activity and correlated with lower viral load in patients. Therefore, we postulate that such mutations with phenotypic consequences of severely reducing viral replication may be a mechanism that results in persistence.

## Conclusions

Our analysis was based on a single snapshot of infection, when a diagnostic sample was taken when patients presented to an Ebola virus treatment centre, with an acute (at the time) undiagnosed febrile illness. What happens to minor variants and the viral population as the infection progresses in humans is unknown. We postulate that the minor variants in hospitalised survivors may act as both defective interfering genomes and produce aberrant or non-functional proteins. Both of these factors may act to lower viral load and push patients with EVD towards a survival outcome.

## Methods

### Sample collection, sequencing and data collection

Sequencing data used in this project was obtained from individual blood samples taken from 134 patients by the European Mobile Laboratory as part of the global response to the Ebola crisis in West Africa between 2013 and 2016. Over 300 samples were initially sequenced. The sample ID for the data used in this analysis, outcome for the patient and EBOV viral load (1/Ct) are summarised in Additional file 1: Table S1. For sequencing of the samples, RNA-seq libraries were prepared from the DNAse-treated total RNA using the Epicentre ScriptSeq v2 RNA-Seq Library Preparation Kit, followed by 10–15 cycles of amplification and purification using AMPure XP beads. Samples from 86.46% of the fatalities and 52.63% of the survivors were amplified with 15 cycles. Each library was quantified using Qubit and the size distribution assessed using the Agilent 2100 Bioanalyzer, and the final libraries were pooled in equimolar ratios. The raw fastq files generated by HiSeq2500 were trimmed to remove Illumina adapter sequences using Cutadapt v1.2.1 [41]. The option “−O 3” was set, so the that 3′ end of any reads which matched the adapter sequence with greater than 3 bp was trimmed off. The reads were further trimmed to remove low-quality bases, using Sickle v1.200 [42] with a minimum window quality score of 20. After trimming, reads shorter than 10 bp were removed.

### Dominant genome generation

Hisat2 v2.1.0 [43] was used to map the trimmed reads on the human reference genome assembly GRCh38 (release-91) downloaded from the Ensembl FTP site. The unmapped reads were extracted by bam2fastq (v1.1.0) and then mapped on a known EBOV genome (GenBank sequence accession: KY426690) using Bowtie2 v2.3.5.1 [43] by setting the options to parameters “--local -X 2000 --no-mixed”, followed by Sam file to Bam file conversion, sorting and removal of the reads with a mapping quality score below 11 using SAMtools v1.9 [44]. After that, the PCR and optical duplicate reads in the bam files were discarded using the MarkDuplicates in the Picard toolkit v2.18.25 (http://broadinstitute.github.io/picard/) with the option of “REMOVE_DUPLICATES=true”. The resultant Bam file was processed by Quasirecomb v1.2 [45] to generate a phred-weighted table of nucleotide frequencies which were parsed with a custom perl script to generate a dominant genome sequence as our previous description [46]. The dominant genome sequence was then used as a template in the second round of mapping to generate a reference genome sequence for all downstream analysis. With this method, we generated reference dominant EBOV genome sequences for each hospitalised fatal cases and hospitalised survival cases.

#### Nucleotide and amino acid deviations

Reads (unmapped on human genome) were aligned to the reference EBOV-dominant genome sequence using Bowtie2 with the parameter of “--local -X 2000 --no-mixed”. The Bowtie2 outputs were processed in the same way as above to generate a Bam file without read duplication. This Bam file was then processed by diversiutils script in DiversiTools (http://josephhughes.github.io/btctools/) with the “-orfs” function to generate the count of transition or transversion deviations and read coverage at each nucleotide site of the alignment and also the number of non-synonymous and synonymous mutations caused by the nucleotide deviation and coverage at each amino acid site in protein. In order to distinguish of low-frequency variants from Illumina sequence errors, the diversiutils used the calling algorithms based on the Illumina quality scores to calculate a *P* value for each variant at each nucleotide site [47]. The outputs of diversiutils were then filtered based on the *P* value (< 0.05) to remove the low-frequency variants from Illumina sequence errors. The site nucleotide variation frequency is the ratio of the nucleotide deviation number to the read coverage at each site of EBOV genome sequences.

#### Statistical analysis

We used the R language v3.5.2 [48] for statistical computations. The normality of the distribution of the data was checked by Shapiro-Wilk *W* test (*P* > 0.05, sample size < 5000) using *shapiro.test* in the “stats” package [48] or by Anderson-Daring test (*P* > 0.05, sample size > 5000) using *ad.test* in the “nortest” package [49]. Student’s *t* test was preformed using *t.test* in the “stats” package [49] to determine if there was a significant difference between the two groups if both of them were normal distributions. Wilcoxon signed rank test preformed with *wilcox.test* in the “stats” package [49] (or the online version at https://astatsa.com/WilcoxonTest/ for the *P* < 2.2e^−16^) with “exact = FALSE” function was an alternative to the *t* test when the two groups of data were not assumed to follow normal distributions. The correlations between nucleotide (or amino acid) deviation frequencies and viral load (1/Ct) were measured by the Spearman rank correlation coefficient, which was calculated and visualised by using *ggscatter* in the “ggplot2” package [50]. The correlation coefficient was comprised between − 1 and + 1, where − 1 indicated a strong negative correlation, 0 meant that there was no association and + 1 indicated a strong positive correlation. Regression line, including a 95% confidence region (light grey coloured), was also added in the correlation scatterplots that showed the overall trend of a set of data. Generalised linear models for Figs. 1e, 2c–e and 5e were fitted using *glm* with variation frequency as a response, and viral load (1/Ct) and outcomes (survivors and fatalities) as predictor variables and interaction between viral load and outcomes, by setting the family function to ‘family = Gamma (link = “inverse”)’. The summaries of the goodness of fit measures are listed in Additional file 1: Table S4.

#### Plasmid construction and production

The EBOV Makona strain mini-genome system used in this study was the same used by Garcia-Dorival et al. [22]. A codon-optimised L gene for expression in human cells was derived from the Makona strain [NCBI sequence reference number KJ660347.2] with either a stop codon at amino acid position 986 (referred as L_STOP_) or with amino acid changes N>S, Q>R and F>S at positions 572, 986 and 2061 (referred as L_3mut_), respectively. These were cloned into the expression vector pUC57. The transcription of the viral gene was under the control of a T7 polymerase promoter. A codon-optimised cDNA sequence for human cell expression encoding the viral protein VP35 was cloned into the pEGFP-N1 vector for the expression of EBOV Makona strain VP35 [NCBI sequence reference number KJ660347.2] with a C-terminal eGFP tag. The Invitrogen GeneArt Gene Synthesis service (Thermo Fisher Scientific) was used for plasmid design, cloning and codon optimisation of the coding sequences. SURE competent cells (Agilent) were used for the production of the mini-genome system plasmids pMG, pL, pVP30, pVP35, pNP, pRLTK, pL_3mut_ and pL_STOP_. Subcloning efficiency DH5α competent cells (Invitrogen) were used to transform peGFP-N1 and peGFP according to the manufacturer’s instructions. For the preparation of the plasmids, the QIAGEN® Plasmid Maxi Kit (Qiagen) was used. For each maxiprep, 0.5 L of turbid overnight culture of plasmid DNA-containing bacteria with the correspondent selective antibiotic was used as input. cDNA plasmids were eluted and resuspended in 300 μl of nuclease-free water. Plasmid stock concentrations were measured by NanoDrop ONE spectrophotometer (Thermo Scientific). Endonuclease digestion was performed with SacI-HF, SalI-HF and BglII-HF (New England Biolab) on plasmid DNA to obtain linear fragments and to determine their size by electrophoresis for screening purposes. The amount of endonuclease was 1 unit/50 μl reaction.

#### Cell culture

BSR-T7 cells, a BHK cell line stably expressing T7 polymerase, were used for the expression of the EBOV mini-genome system and the EBOV VP35-eGFP co-immunoprecipitation assays. BSR-T7 cells were cultured in Dulbecco’s modified Eagle’s medium (DMEM; Sigma-Aldrich) supplemented with 10% foetal bovine serum (FBS; Sigma-Aldrich), 1% [v/v] GlutaMAX, 1% [v/v] penicillin-streptomycin and 0.6% [v/v] Geneticin® G418 (Thermo Fisher) at 37 °C with 5% CO_2_. Note that this cell line was not authenticated, but the expression of T7 was confirmed as part of the study.

#### EBOV transcription/replication mini-genome system

In order to measure the functionality of EBOV L_STOP_, L_3mut_ and EBOV VP35/eGFP, an EBOV mini-genome system was used [22]. Unless otherwise indicated, transfection of the plasmids was carried out with Lipofectamine™ 2000 (Invitrogen) at the following cDNA amounts per well in a 24-well plate format: 500 ng pMG, 250 ng pNP, 125 ng pVP30, 125 ng pVP35, 125 ng pL and 250 ng pRLTK. Addition of pL_STOP_, pL_3mut_, pVP35-eGFP and pUC57 cDNA amounts is indicated in each experimental set. For the mini-genome system experiments, 10^5^ cells/well were seeded 24 h prior to plasmid transfection to obtain 90% confluence. Cells were lysed 24 h post-transfection with 100 μl of 5X Passive Lysis Buffer (PLB; Promega) diluted fivefold with water. For the measurement of the mini-genome system’s activity, a Dual-Luciferase® Reporter assay (DLR™; Promega) was performed, where 100 μl of Luciferase Assay Reagent II and 100 μl of Stop&Glo® reagent (Promega) were used per 20 μl of supernatant of cell lysate in a 96-well white plate. *Firefly* luciferase and *Renilla* luciferase activities were read in a GloMax® Explorer plate reader (Promega). The *Renilla* luciferase activity measurements were used to normalise the *Firefly* luciferase activity of the samples. Each condition was done in triplicate from different samples for a two-tailed unpaired *t* test statistical analysis.

#### Co-immunoprecipitation of VP35-eGFP

Calcium phosphate transfection was used as the plasmid transfection method in the co-immunoprecipitation assays. To ensure 50–60% cell confluency, two 10-cm^2^ plates per experimental condition were seeded with 1.5 × 10^6^ BSR-T7 cells 24 h prior to transfection. Each plate was transfected with 20 μg of the total amount of plasmid (17.5 μg pMG, 9 μg pNP, 4.5 μg pVP30, 4.5 μg pPV35-eGFP or peGFP, 4.5 μg pL or pL_STOP_) together with 61 μl 2 M CaCl_2_, 500 μl HEPES-buffered saline 2× (274 mM NaCl; 10 mM KCl; 1.4 mM Na_2_HPO_4_, pH 7.05) and 399 μl H_2_0. Plates were incubated for 48 h at 37 °C and harvested, and cell pellets were washed in PBS. Cell pellets were lysed in 200 μl of lysis buffer (10 mM Tris/HCl, pH 7.5; 150 mM NaCl; 0.5 mM EDTA; 0.5% [v/v] NP-40 supplemented with 1X Halt protease Inhibitor Cocktail EDTA-Free (Thermo Scientific)), incubated on ice for 30 min, cleared by centrifugation at 14,000*g* for 10 min at 4 °C and diluted fivefold with dilution/wash buffer (10 mM Tris-Cl, pH 7.5; 150 mM NaCl; 0.5 mM EDTA supplemented with 1X Halt Protease Inhibitor Cocktail EDTA-Free). The GFP-Trap® agarose beads (ChromoTek) were equilibrated with ice-cold dilution/wash buffer and incubated with the diluted cell lysates on a rotary mixer at 4 °C overnight, and then centrifuged at 2500*g* for 2 min. The pellet was washed two times with dilution/wash buffer, and the beads were resuspended with 100 μl 2X SDS buffer (1 M Tris, pH 6.8; 50% [v/v] glycerol; 10% [w/v] SDS; 0.5% [v/v] bromophenol blue; 0.5% [v/v] β-mercaptoethanol).

#### Western blot

The Pierce™ BCA Protein Assay Kit (Thermo Scientific) was used for quantification of total protein in the mini-genome system cell lysates to equal the protein concentration between samples. Protein samples were boiled and denatured for 10 min at 95 °C with 4X Laemmli sample buffer (Bio-Rad), and 2.5 μl of total protein was loaded in 10% polyacrylamide SDS-PAGE gels for immunoblotting and run with 1X SDS-PAGE running buffer (25 mM Tris-HCl, pH 8.3; 250 mM glycine, 0.1% [w/v] SDS) at 100–120 V, 400 mA for approximately 1.5 h. Transfer was performed by using a Bio-Rad semi-dry apparatus at 25 V for 30 min to 1 h with Towbin buffer (25 mM Tris-base, pH 8.1–8.5; 192 mM glycine; 20% [v/v] methanol) depending on the protein of interest’s size. After transfer, the membranes were blocked in 10% [w/v] skimmed milk powder (Marvel) made up in Tris-buffered saline (0.25 M Tris-base, pH 7.4; 1.5 M NaCl) containing 10% [v/v] Tween-20 (TBS-T). Washes were performed with TBS-T. Primary antibodies in 5% [w/v] skimmer milk powder TBS-T ware added to the membranes and incubated overnight at 4 °C on a rocker. Antibodies used for the primary incubation were anti-EBOV-L (IBT Bioservices; 0301-045), EBOV-NP (IBT Bioservices, 0301-012), EBOV-VP35 (IBT Bioservices, 0301-040), anti-eGFP (Santa Cruz, sc-8334), anti-*Firefly* luciferase (Abcam, ab185923) and anti-GAPDH (Abcam, ab8245). Horseradish peroxidase-conjugated secondary antibody anti-mouse (Sigma, A4416) or anti-rabbit (Sigma, A6154) was diluted in TBS-T 5% [w/v] skimmed milk, was added to the membrane and was incubated for 1 h at room temperature. Protein-antibody complexes were visualised using the Clarity™ Western ECL Blotting Substrates (Bio-Rad). A ChemiDoc Touch Gel Imaging System (Bio-Rad) was utilised to obtain pictures of the developed membranes.

## Supplementary information

**Additional file 1: Supplementary figures S1-S7 and tables S1-S4.**

**Additional file 2.** Review history.

## Data Availability

All viral sequence data used in this analysis were deposited with the NCBI under BioProject Accession Number PRJNA577693 and can be accessed at https://www.ncbi.nlm.nih.gov/bioproject/?term=PRJNA577693 [51].
