# Supplementary material for: Variation around the dominant viral genome sequence contributes to viral load and outcome in patients with Ebola virus disease
Source: Genome Biol. 2020 Sep 7;21:238. doi: 10.1186/s13059-020-02148-3 (PMC7475720; doi:10.1186/s13059-020-02148-3)
Supplement: Supplementary file 1 — Additional file 1: Supplementary figures S1-S7 and tables S1-S4. [file 13059_2020_2148_MOESM1_ESM.docx]

**Additional File 1**

Fig. S1: The ratio of average frequency of transversions to average frequency of transitions along the EBOV genome in windows slide of 200 nts.

Fig. S2: Correlation between frequency difference and P-value of each protein site.

Fig. S3. The percentage of top three codons.

Fig. S4. Uncropped western blots assessing the EBOV mini-genome system relative activity at different ratios between EBOV L and LSTOP.

Fig. S5. Uncropped western blots assessing the EBOV mini-genome system relative activity at equal EBOV L but different LSTOP amounts.

Fig. S6. Uncropped western blots assessing the functionality in an EBOV mini-genome system assay of the L3mut protein at different ratios with EBOV L.

Fig. S7. Uncropped western blots of the co-immunoprecipitation (coIP) assay fractions to examine the interaction between EBOV VP35 with EBOV LSTOP.

Table S1: General statistics of PCR cycle, average coverage, variation coefficient, number of SNPs, average frequency of SNPs and viral load (1/Ct) for each sample.

Table S2: Coefficients generated by glms.

Table S3: Count of transition (Ts) and transversion (Tv) in all samples.

Table S4: Summary of the goodness of fit measures.


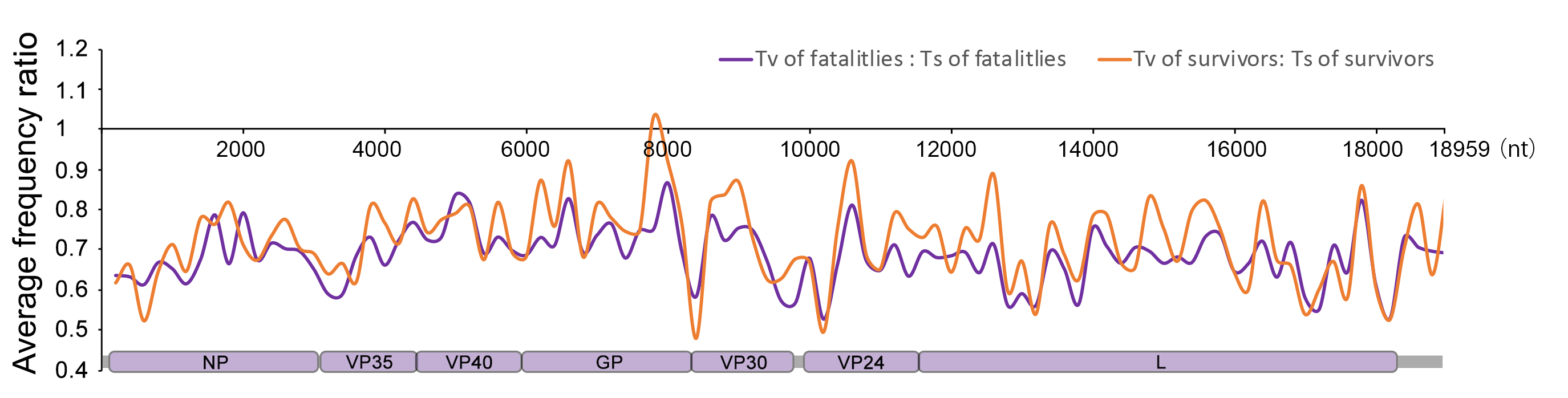


**Fig. S1.** The ratio of average frequency of transversions to average frequency of transitions along the EBOV genome in windows slide of 200 nts. Ratio > 1 suggests the transversion is more frequent than transition at this region.


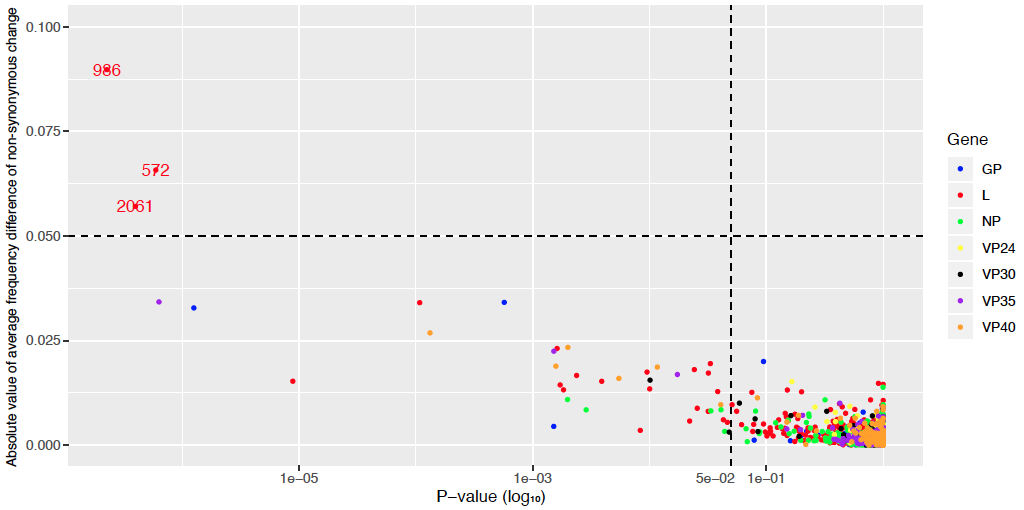

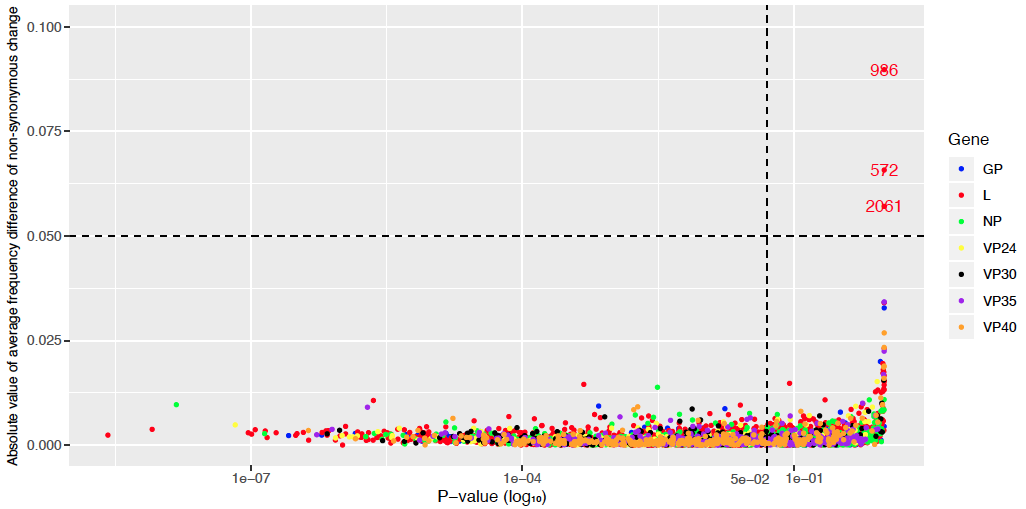


(a)

(b)

**Fig. S2.** Correlation between frequency difference and P-value of each protein site. (a) Absolute value of average frequency difference of non-synonymous change between hospitalised survivors and hospitalised fatalities plotting against P-values, calculated by with a one-sided Wilcoxon rank sum test to test if the non-synonymous nucleotide substitution frequencies of survivors was greater than fatalities at each protein site. (b) Absolute value of average frequency difference of non-synonymous change between hospitalised survivors and hospitalised fatalities plotting against P-values calculated by with a one-sided Wilcoxon rank sum test to test if the non-synonymous nucleotide substitution frequencies of survivors was less than fatalities at each protein site. L protein at positions 572, 986 and 2061 were labeled in both (a) and (b).


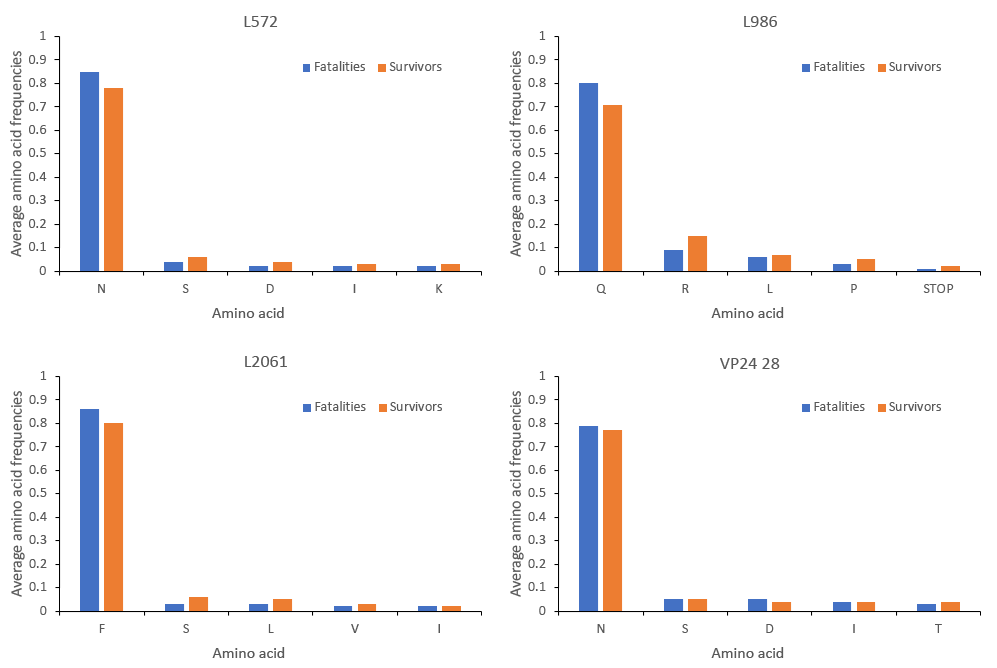


**Fig. S3.** The percentage of top three codons in 96 hospitalised fatal and 38 hospitalised survivor cases at positions 527, 986 and 2061 in the L protein and position 28 in the VP24.


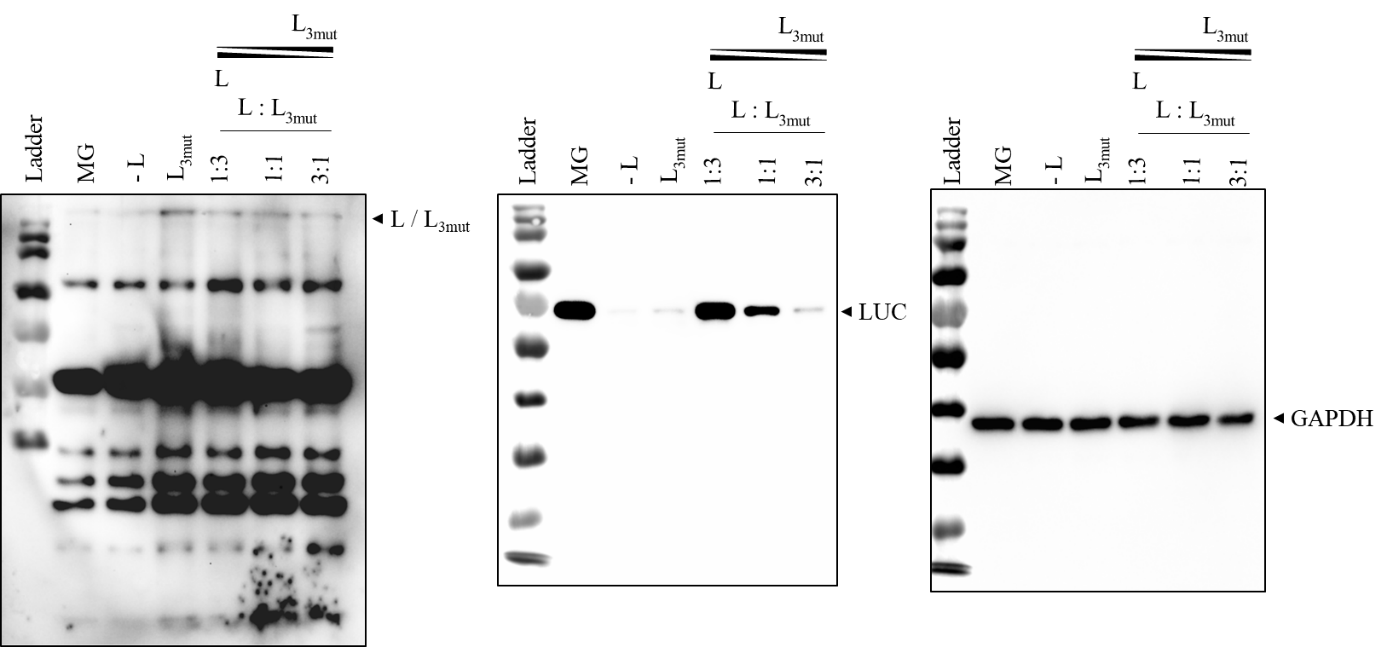


**Fig. S4.** Uncropped western blots assessing the EBOV mini-genome system relative activity at different ratios between EBOV L and L_STOP_.


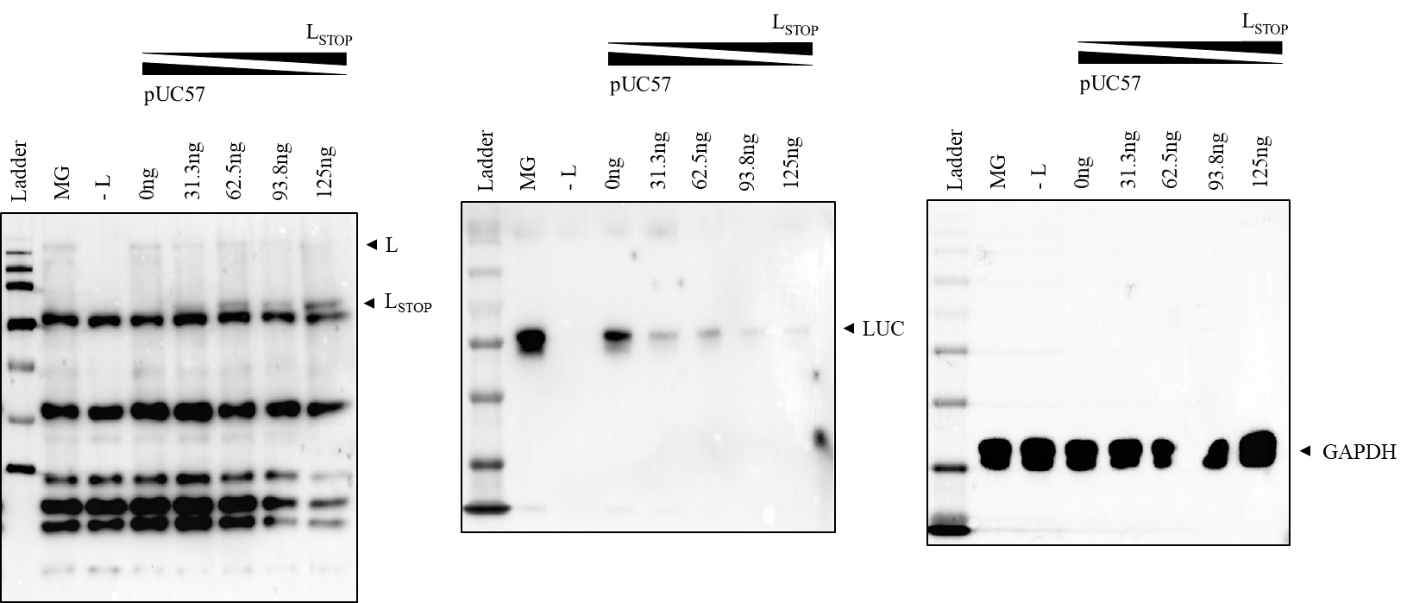


**Fig. S5**. Uncropped western blots assessing the EBOV mini-genome system relative activity at equal EBOV L but different L_STOP_ amounts.


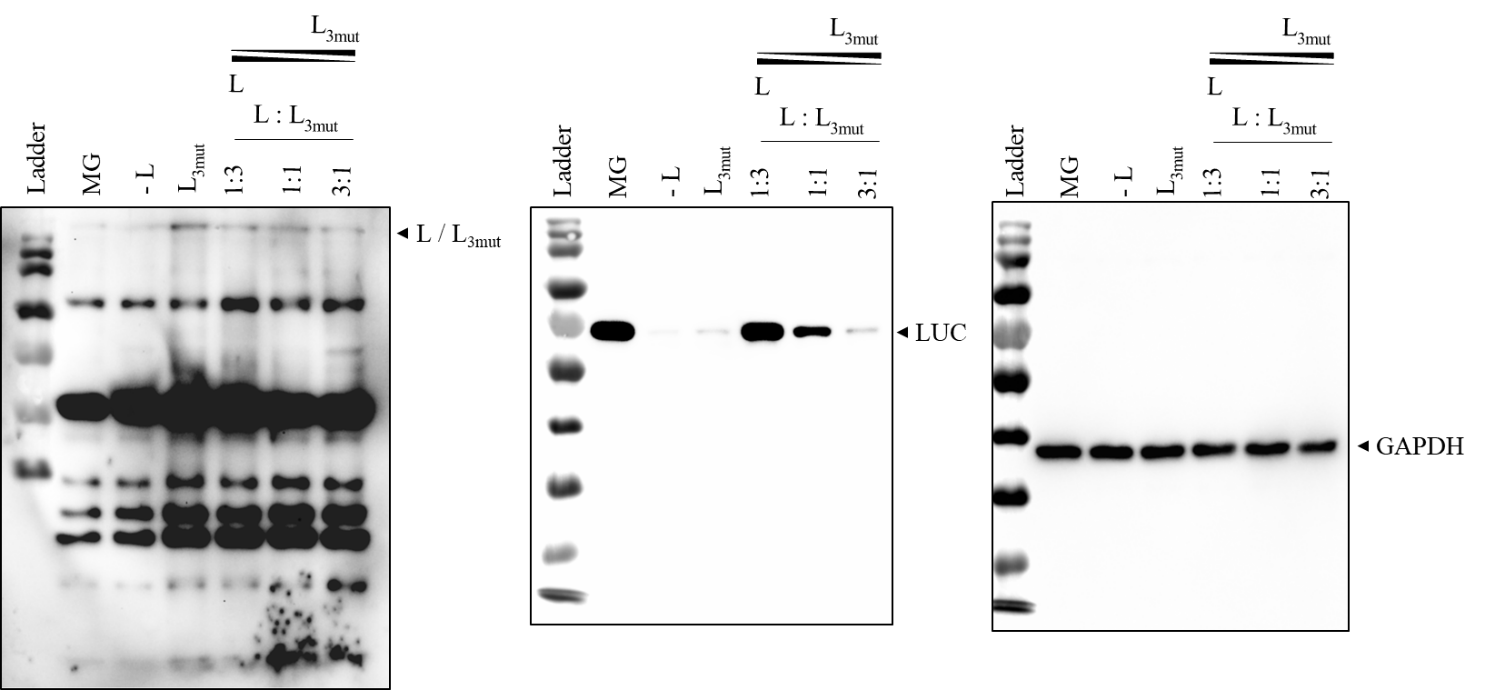


**Fig. S6.** Uncropped western blots assessing the functionality in an EBOV mini-genome system assay of the L_3mut_ protein at different ratios with EBOV L.


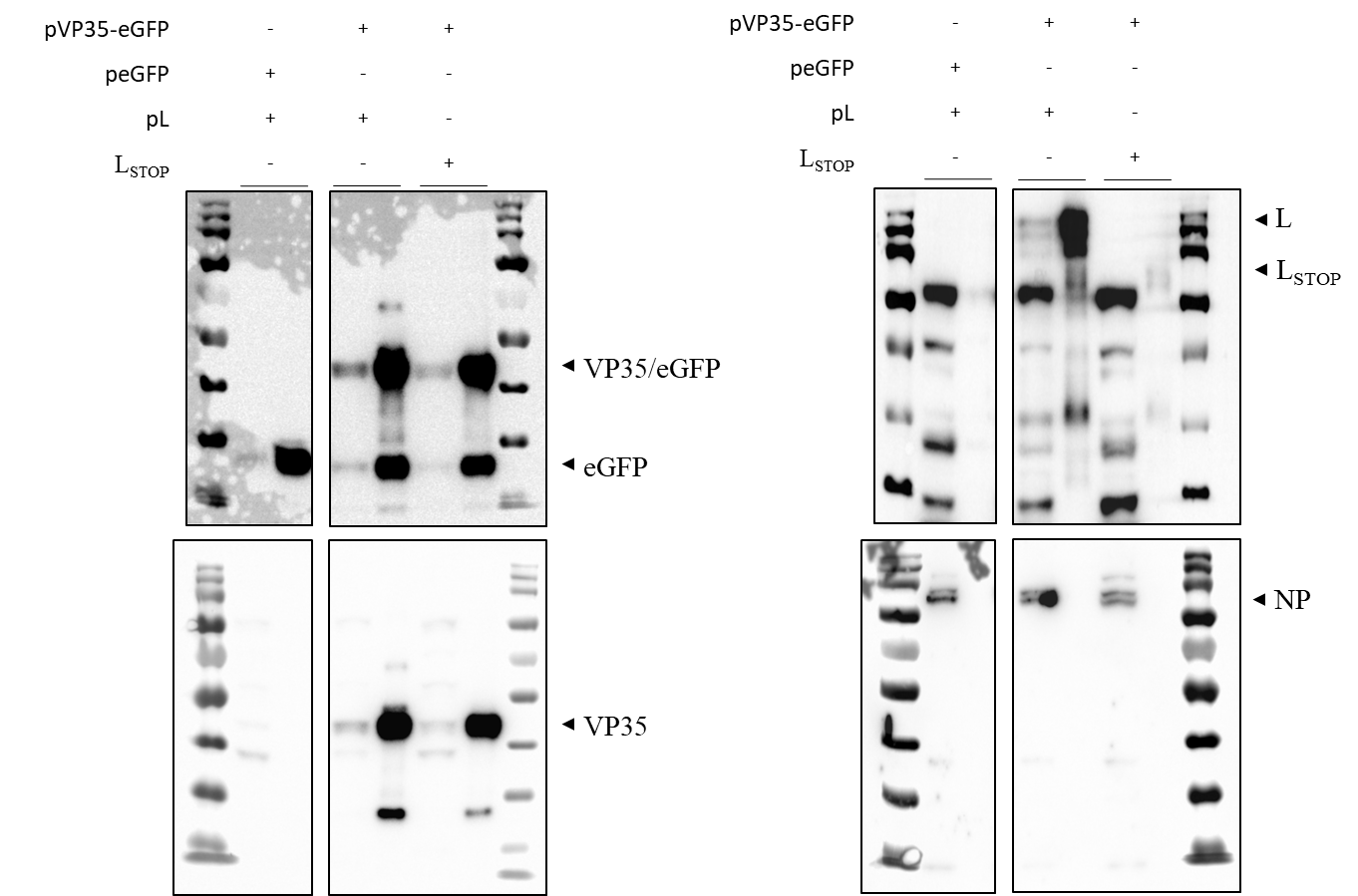


**Fig. S7.** Uncropped western blots of the co-immunoprecipitation (coIP) assay fractions to examine the interaction between EBOV VP35 with EBOV L_STOP_.

**Table S1:** General statistics of PCR cycle, average coverage, variation coefficient, number of SNPs, average frequency of SNPs and viral load (1/Ct) for each sample. The mean and standard deviation of these factors were also calculated for both fatalities and survivors.

| **Sample ID** | **PCR cycle** | **Average coverage** | **Variation coefficient** | **Number of SNPs** | **Average frequency of SNPs** | **Viral load (1/Ct)** |
| --- | --- | --- | --- | --- | --- | --- |
| Fatality-1 | 15 | 654.57 | 0.45 | 24287 | 1.96E-03 | 5.80E-02 |
| Fatality-2 | 15 | 3656.46 | 0.35 | 160103 | 2.31E-03 | 7.16E-02 |
| Fatality-3 | 15 | 75.05 | 0.78 | 4302 | 3.03E-03 | 4.39E-02 |
| Fatality-4 | 15 | 6079.84 | 0.30 | 275486 | 2.39E-03 | 6.84E-02 |
| Fatality-5 | 15 | 1427.53 | 0.43 | 62578 | 2.31E-03 | 5.86E-02 |
| Fatality-6 | 15 | 413.63 | 0.62 | 23375 | 2.98E-03 | 5.63E-02 |
| Fatality-7 | 15 | 438.13 | 0.43 | 16858 | 2.03E-03 | 6.31E-02 |
| Fatality-8 | 15 | 640.08 | 0.37 | 23869 | 1.97E-03 | 6.51E-02 |
| Fatality-9 | 15 | 566.73 | 0.45 | 24772 | 2.31E-03 | 6.64E-02 |
| Fatality-10 | 15 | 1568.12 | 0.39 | 59808 | 2.01E-03 | 6.25E-02 |
| Fatality-11 | 15 | 79.61 | 0.55 | 3754 | 2.50E-03 | 5.33E-02 |
| Fatality-12 | 15 | 169.37 | 0.53 | 7232 | 2.26E-03 | 5.39E-02 |
| Fatality-13 | 15 | 39.98 | 0.64 | 5913 | 7.83E-03 | 6.33E-02 |
| Fatality-14 | 15 | 371.38 | 0.47 | 15800 | 2.25E-03 | 5.14E-02 |
| Fatality-15 | 15 | 4306.12 | 0.30 | 175077 | 2.14E-03 | 7.14E-02 |
| Fatality-16 | 15 | 3492.66 | 0.35 | 145661 | 2.20E-03 | 6.98E-02 |
| Fatality-17 | 15 | 10351.52 | 0.26 | 444520 | 2.27E-03 | 7.34E-02 |
| Fatality-18 | 15 | 2025.2 | 0.43 | 80581 | 2.10E-03 | 6.84E-02 |
| Fatality-19 | 15 | 1261.04 | 0.41 | 57948 | 2.42E-03 | 5.40E-02 |
| Fatality-20 | 15 | 944.01 | 0.41 | 40090 | 2.24E-03 | 5.79E-02 |
| Fatality-21 | 15 | 7825.25 | 0.28 | 287549 | 1.94E-03 | 7.95E-02 |
| Fatality-22 | 15 | 816.98 | 0.43 | 97979 | 6.33E-03 | 5.24E-02 |
| Fatality-23 | 15 | 3262.9 | 0.35 | 139813 | 2.26E-03 | 6.21E-02 |
| Fatality-24 | 15 | 2880.43 | 0.33 | 122159 | 2.24E-03 | 6.39E-02 |
| Fatality-25 | 15 | 3874.44 | 0.32 | 147584 | 2.01E-03 | 7.22E-02 |
| Fatality-26 | 15 | 1670.33 | 0.37 | 67290 | 2.12E-03 | 5.63E-02 |
| Fatality-27 | 15 | 9950.13 | 0.28 | 359825 | 1.91E-03 | 7.35E-02 |
| Fatality-28 | 15 | 1559.77 | 0.38 | 61504 | 2.08E-03 | 5.95E-02 |
| Fatality-29 | 15 | 1350.52 | 0.38 | 55736 | 2.18E-03 | 5.71E-02 |
| Fatality-30 | 15 | 648.9 | 0.41 | 29936 | 2.44E-03 | 5.42E-02 |
| Fatality-31 | 15 | 1454.27 | 0.44 | 60962 | 2.21E-03 | 6.20E-02 |
| Fatality-32 | 15 | 2936.81 | 0.36 | 195692 | 3.51E-03 | 6.71E-02 |
| Fatality-33 | 15 | 1443.69 | 0.41 | 61540 | 2.25E-03 | 5.23E-02 |
| Fatality-34 | 15 | 938.24 | 0.49 | 45831 | 2.58E-03 | 5.45E-02 |
| Fatality-35 | 15 | 3258.84 | 0.38 | 139726 | 2.26E-03 | 7.11E-02 |
| Fatality-36 | 15 | 1546.01 | 0.37 | 56948 | 1.94E-03 | 6.43E-02 |
| Fatality-37 | 15 | 748.51 | 0.46 | 29324 | 2.07E-03 | 5.54E-02 |
| Fatality-38 | 15 | 1075.11 | 0.42 | 44042 | 2.16E-03 | 5.75E-02 |
| Fatality-39 | 15 | 323.65 | 0.46 | 12602 | 2.05E-03 | 5.89E-02 |
| Fatality-40 | 15 | 1607.18 | 0.39 | 59090 | 1.94E-03 | 6.55E-02 |
| Fatality-41 | 15 | 595.84 | 0.46 | 31873 | 2.82E-03 | 5.70E-02 |
| Fatality-42 | 15 | 656.29 | 0.44 | 35591 | 2.86E-03 | 5.90E-02 |
| Fatality-43 | 15 | 1310.29 | 0.38 | 49538 | 1.99E-03 | 6.32E-02 |
| Fatality-44 | 15 | 382.11 | 0.43 | 21071 | 2.91E-03 | 5.27E-02 |
| Fatality-45 | 15 | 207.61 | 0.49 | 11921 | 3.03E-03 | 5.34E-02 |
| Fatality-46 | 15 | 122.05 | 0.72 | 7166 | 3.10E-03 | 5.00E-02 |
| Fatality-47 | 15 | 606.82 | 0.44 | 24288 | 2.11E-03 | 6.19E-02 |
| Fatality-48 | 15 | 220.33 | 0.54 | 9790 | 2.34E-03 | 5.87E-02 |
| Fatality-49 | 15 | 69.67 | 0.54 | 3318 | 2.52E-03 | 5.10E-02 |
| Fatality-50 | 15 | 460.3 | 0.45 | 29349 | 3.36E-03 | 5.29E-02 |
| Fatality-51 | 15 | 16.16 | 0.84 | 856 | 2.80E-03 | 3.90E-02 |
| Fatality-52 | 15 | 238.36 | 0.47 | 10349 | 2.29E-03 | 4.87E-02 |
| Fatality-53 | 15 | 717.49 | 0.41 | 33096 | 2.44E-03 | 5.84E-02 |
| Fatality-54 | 15 | 1856.85 | 0.37 | 71788 | 2.04E-03 | 7.09E-02 |
| Fatality-55 | 15 | 260.7 | 0.54 | 11304 | 2.29E-03 | 5.44E-02 |
| Fatality-56 | 15 | 99.07 | 0.74 | 4937 | 2.64E-03 | 4.69E-02 |
| Fatality-57 | 15 | 1083.26 | 0.39 | 48203 | 2.35E-03 | 5.76E-02 |
| Fatality-58 | 15 | 410.21 | 0.64 | 16407 | 2.11E-03 | 6.12E-02 |
| Fatality-59 | 15 | 606.7 | 0.46 | 33171 | 2.89E-03 | 5.64E-02 |
| Fatality-60 | 15 | 234.8 | 0.47 | 11003 | 2.47E-03 | 4.69E-02 |
| Fatality-61 | 15 | 947.96 | 0.40 | 41410 | 2.31E-03 | 5.78E-02 |
| Fatality-62 | 15 | 208.33 | 0.72 | 9151 | 2.32E-03 | 5.70E-02 |
| Fatality-63 | 15 | 325.31 | 0.46 | 14969 | 2.43E-03 | 4.80E-02 |
| Fatality-64 | 15 | 854.78 | 0.46 | 33962 | 2.10E-03 | 7.21E-02 |
| Fatality-65 | 15 | 2348.65 | 0.34 | 90787 | 2.04E-03 | 7.22E-02 |
| Fatality-66 | 15 | 332.04 | 1.24 | 14658 | 2.33E-03 | 6.21E-02 |
| Fatality-67 | 15 | 1953.61 | 0.36 | 105431 | 2.85E-03 | 6.13E-02 |
| Fatality-68 | 15 | 130.26 | 0.55 | 5381 | 2.18E-03 | 5.52E-02 |
| Fatality-69 | 15 | 318.94 | 0.51 | 14044 | 2.32E-03 | 4.90E-02 |
| Fatality-70 | 15 | 88.31 | 1.27 | 4274 | 2.56E-03 | 5.56E-02 |
| Fatality-71 | 15 | 362.58 | 0.86 | 16732 | 2.44E-03 | 5.98E-02 |
| Fatality-72 | 15 | 4085.07 | 0.35 | 156742 | 2.02E-03 | 8.21E-02 |
| Fatality-73 | 15 | 656.62 | 0.46 | 29004 | 2.33E-03 | 5.60E-02 |
| Fatality-74 | 15 | 90.9 | 0.55 | 3495 | 2.03E-03 | 5.72E-02 |
| Fatality-75 | 15 | 495.76 | 0.59 | 17483 | 1.86E-03 | 6.38E-02 |
| Fatality-76 | 15 | 2783.9 | 0.35 | 104962 | 1.99E-03 | 6.85E-02 |
| Fatality-77 | 15 | 413.76 | 0.46 | 16423 | 2.10E-03 | 6.06E-02 |
| Fatality-78 | 15 | 1049.88 | 0.39 | 38869 | 1.95E-03 | 7.38E-02 |
| Fatality-79 | 15 | 36.34 | 0.59 | 1487 | 2.16E-03 | 4.38E-02 |
| Fatality-80 | 15 | 1579.16 | 0.37 | 63657 | 2.13E-03 | 6.04E-02 |
| Fatality-81 | 15 | 137.89 | 0.71 | 5778 | 2.22E-03 | 5.42E-02 |
| Fatality-82 | 15 | 427.77 | 0.59 | 18199 | 2.24E-03 | 6.41E-02 |
| Fatality-83 | 15 | 614.38 | 0.45 | 23336 | 2.01E-03 | 6.73E-02 |
| Fatality-84 | 12 | 663.67 | 0.49 | 38357 | 3.05E-03 | 5.51E-02 |
| Fatality-85 | 12 | 132.94 | 0.50 | 6773 | 2.69E-03 | 4.16E-02 |
| Fatality-86 | 12 | 1341.63 | 0.42 | 56471 | 2.22E-03 | 5.75E-02 |
| Fatality-87 | 12 | 36.4 | 0.78 | 1942 | 2.82E-03 | 4.01E-02 |
| Fatality-88 | 12 | 36.01 | 0.55 | 1753 | 2.58E-03 | 4.66E-02 |
| Fatality-89 | 12 | 57.98 | 0.55 | 2604 | 2.37E-03 | 4.89E-02 |
| Fatality-90 | 12 | 116.18 | 0.70 | 5317 | 2.42E-03 | 4.83E-02 |
| Fatality-91 | 12 | 48.03 | 1.06 | 2328 | 2.58E-03 | 4.60E-02 |
| Fatality-92 | 12 | 82.8 | 0.56 | 4110 | 2.62E-03 | 4.57E-02 |
| Fatality-93 | 12 | 434.72 | 0.45 | 20365 | 2.47E-03 | 4.93E-02 |
| Fatality-94 | 12 | 122.33 | 0.50 | 5855 | 2.53E-03 | 4.99E-02 |
| Fatality-95 | 12 | 77.48 | 0.56 | 3858 | 2.64E-03 | 4.62E-02 |
| Fatality-96 | 12 | 112.07 | 0.56 | 5509 | 2.60E-03 | 4.66E-02 |
|  |  |  |  |  |  |  |
| Survivor-1 | 15 | 59.68 | 0.57 | 2600 | 2.30E-03 | 5.26E-02 |
| Survivor-2 | 15 | 94.35 | 0.71 | 3952 | 2.21E-03 | 5.23E-02 |
| Survivor-3 | 15 | 607.95 | 0.43 | 26571 | 2.31E-03 | 5.15E-02 |
| Survivor-4 | 15 | 356.22 | 0.52 | 17468 | 2.59E-03 | 5.06E-02 |
| Survivor-5 | 15 | 101 | 0.49 | 4806 | 2.51E-03 | 4.52E-02 |
| Survivor-6 | 15 | 1846.14 | 0.37 | 81529 | 2.33E-03 | 5.46E-02 |
| Survivor-7 | 15 | 96.92 | 0.60 | 4626 | 2.52E-03 | 4.51E-02 |
| Survivor-8 | 15 | 350.38 | 0.51 | 14552 | 2.19E-03 | 5.09E-02 |
| Survivor-9 | 15 | 42.7 | 0.93 | 3138 | 3.88E-03 | 4.54E-02 |
| Survivor-10 | 15 | 116.72 | 0.59 | 9498 | 4.30E-03 | 4.68E-02 |
| Survivor-11 | 15 | 63.61 | 0.62 | 4509 | 3.75E-03 | 4.67E-02 |
| Survivor-12 | 15 | 26.49 | 0.60 | 1207 | 2.41E-03 | 4.73E-02 |
| Survivor-13 | 15 | 172.37 | 0.56 | 8101 | 2.48E-03 | 5.03E-02 |
| Survivor-14 | 15 | 64.62 | 0.56 | 2984 | 2.44E-03 | 4.55E-02 |
| Survivor-15 | 15 | 127.42 | 0.49 | 7616 | 3.16E-03 | 5.19E-02 |
| Survivor-16 | 15 | 159.21 | 0.51 | 9256 | 3.07E-03 | 4.32E-02 |
| Survivor-17 | 15 | 54.12 | 0.63 | 3182 | 3.11E-03 | 3.78E-02 |
| Survivor-18 | 15 | 257.21 | 0.48 | 9888 | 2.03E-03 | 5.79E-02 |
| Survivor-19 | 15 | 69.04 | 0.63 | 3094 | 2.37E-03 | 4.55E-02 |
| Survivor-20 | 15 | 20.76 | 0.79 | 1084 | 2.76E-03 | 4.40E-02 |
| Survivor-21 | 11 | 163.58 | 0.57 | 8127 | 2.62E-03 | NA |
| Survivor-22 | 11 | 49.64 | 0.90 | 2145 | 2.29E-03 | 4.84E-02 |
| Survivor-23 | 11 | 320.75 | 0.46 | 13045 | 2.15E-03 | 5.09E-02 |
| Survivor-24 | 11 | 61.96 | 0.56 | 3039 | 2.60E-03 | 4.34E-02 |
| Survivor-25 | 12 | 286.1 | 0.64 | 16974 | 3.13E-03 | 5.75E-02 |
| Survivor-26 | 12 | 26.18 | 0.69 | 1311 | 2.66E-03 | 4.14E-02 |
| Survivor-27 | 12 | 59.6 | 0.62 | 3026 | 2.68E-03 | 4.57E-02 |
| Survivor-28 | 12 | 136.49 | 0.52 | 11119 | 4.30E-03 | NA |
| Survivor-29 | 12 | 114.41 | 0.57 | 5492 | 2.54E-03 | 3.17E-02 |
| Survivor-30 | 12 | 87.82 | 0.59 | 4904 | 2.95E-03 | 4.30E-02 |
| Survivor-31 | 12 | 73.89 | 0.52 | 3495 | 2.50E-03 | 4.83E-02 |
| Survivor-32 | 12 | 70.19 | 0.53 | 3494 | 2.64E-03 | 4.79E-02 |
| Survivor-33 | 12 | 130.99 | 0.50 | 6286 | 2.53E-03 | 4.91E-02 |
| Survivor-34 | 12 | 101.4 | 0.60 | 4701 | 2.45E-03 | 4.60E-02 |
| Survivor-35 | 12 | 100.26 | 0.53 | 4495 | 2.37E-03 | 4.90E-02 |
| Survivor-36 | 12 | 49.63 | 0.54 | 2431 | 2.59E-03 | 4.90E-02 |
| Survivor-37 | 12 | 118 | 0.54 | 5770 | 2.59E-03 | 4.72E-02 |
| Survivor-38 | 12 | 198.74 | 0.48 | 9002 | 2.39E-03 | 4.98E-02 |
|  |  |  |  |  |  |  |
| Mean of fatalities | - | 1264.50 | 0.50 | 53933.76 | 2.46E-03 | 5.83E-02 |
| Standard deviation of fatalities | - | 1873.98 | 0.18 | 76115.43 | 7.65E-04 | 8.92E-03 |
| Mean of survivors | - | 956.93 | 0.52 | 41090.73 | 2.53E-03 | 5.54E-02 |
| Standard deviation of survivors | - | 301.56 | 0.11 | 13263.18 | 5.47E-04 | 5.00E-03 |

**Table S2:** Coefficients generated by glms. In each model, the term of “(Intercept)” is the intercept of regression for fatalities and the term of “1/Ct” is the slope of regression for fatalities; The term of “Survivors” indicates the difference of intercept in the regression of hospitalised survivors to hospitalised fatal cases and the term of “1/Ct:Survivors” indicates the difference of slope in the regression of hospitalised survivors to hospitalised fatal cases.

|  | **Term** | **Estimate** | **Std.error** | **Statistic** | **P-value** | |
| --- | --- | --- | --- | --- | --- | --- |
| Deviation (fig. 1e) | (Intercept) | 230.965686 | 77.3391353 | 2.98640119 | 3.38E-03 | ** |
|  | 1/Ct | 3146.1085 | 1339.09337 | 2.34943177 | 2.03E-02 | * |
|  | Survivors | 1.88587439 | 183.898241 | 0.01025499 | 9.92E-01 |  |
|  | 1/Ct:Survivors | 67.2037623 | 3763.07157 | 0.01785875 | 9.86E-01 |  |
| Ts (fig. 1e) | (Intercept) | 440.0658323 | 55.1872552 | 7.97404818 | 7.43E-13 | *** |
|  | 1/Ct | 4405.309702 | 952.291535 | 4.62600952 | 9.00E-06 | *** |
|  | Survivors | -15.75466511 | 132.374611 | -0.1190158 | 9.05E-01 |  |
|  | 1/Ct:Survivors | 426.3647915 | 2706.62585 | 0.15752631 | 8.75E-01 |  |
| Tv (fig. 1e) | (Intercept) | 459.5975569 | 370.122283 | 1.24174517 | 2.17E-01 |  |
|  | 1/Ct | 9657.982575 | 6440.6345 | 1.49953899 | 1.36E-01 |  |
|  | Survivors | 47.9646623 | 869.275996 | 0.05517771 | 9.56E-01 |  |
|  | 1/Ct:Survivors | -567.8873235 | 17808.5584 | -0.0318884 | 9.75E-01 |  |
| L572 (fig. 2c) | (Intercept) | -6.236575332 | 1.19098349 | -5.2364919 | 6.53E-07 | *** |
|  | 1/Ct | 232.4688404 | 22.3129256 | 10.4185728 | 8.78E-19 | *** |
|  | Survivors | 7.068757752 | 2.34627066 | 3.01276314 | 3.12E-03 | ** |
|  | 1/Ct:Survivors | -151.1440822 | 48.5028195 | -3.1161917 | 2.26E-03 | ** |
| L986. (fig. 2d) | (Intercept) | -4.338988089 | 0.97153305 | -4.4661251 | 1.73E-05 | *** |
|  | 1/Ct | 166.2863538 | 18.1421132 | 9.1657654 | 1.04E-15 | *** |
|  | Survivors | 6.433032596 | 1.97841566 | 3.25160821 | 1.47E-03 | ** |
|  | 1/Ct:Survivors | -139.1093669 | 40.594168 | -3.4268313 | 8.22E-04 | *** |
| L2061. (fig. 2e) | (Intercept) | -4.891539849 | 1.21786462 | -4.016489 | 1.00E-04 | *** |
|  | 1/Ct | 216.2778233 | 22.4580962 | 9.63028306 | 7.66E-17 | *** |
|  | Survivors | 7.355973457 | 2.47208468 | 2.97561549 | 3.50E-03 | ** |
|  | 1/Ct:Survivors | -159.3288384 | 50.7223188 | -3.1411978 | 2.09E-03 | ** |
| Stop codon (fig. 3d) | (Intercept) | -75.4304652 | 33.5054089 | -2.2512922 | 2.64E-02 | * |
|  | 1/Ct | 2355.347737 | 611.246093 | 3.85335426 | 2.01E-04 | *** |
|  | Survivors | 120.6005535 | 57.9106187 | 2.08252918 | 3.97E-02 | * |
|  | 1/Ct:Survivors | -2576.836602 | 1150.31965 | -2.2401048 | 2.72E-02 | * |
|  |  |  |  |  |  |  |

Signif. codes: 0 ‘***’ 0.001 ‘**’ 0.01 ‘*’ 0.05 ‘.’ 0.1 ‘ ’ 1

**Table S3:** Count of transition (Ts) and transversion (Tv) in all samples.

| Mutation type | Interchanges | Survivors | | Fatalities | | All samples | |
| --- | --- | --- | --- | --- | --- | --- | --- |
|  |  | Number | % mutates | Number | % mutates | Number | % mutates |
| Ts | A to G | 81765 | 24.85% | 1388631 | 26.79% | 1470396 | 26.67% |
|  | G to A | 22788 | 6.92% | 355847 | 6.87% | 378635 | 6.87% |
|  | C to T | 31722 | 9.64% | 512255 | 9.88% | 543977 | 9.87% |
|  | T to C | 57330 | 17.42% | 910139 | 17.56% | 967469 | 17.55% |
|  | Total | 193605 | 58.83% | 3166872 | 61.10% | 3360477 | 60.96% |
|  |  |  |  |  |  |  |  |
| TV | A to C | 24187 | 7.35% | 390291 | 7.53% | 414478 | 7.52% |
|  | C to A | 19972 | 6.07% | 279580 | 5.39% | 299552 | 5.43% |
|  | G to T | 15755 | 4.79% | 227470 | 4.39% | 243225 | 4.41% |
|  | T to G | 18278 | 5.55% | 258838 | 4.99% | 277116 | 5.03% |
|  | A to T | 23785 | 7.23% | 388049 | 7.49% | 411834 | 7.47% |
|  | T to A | 17137 | 5.21% | 247559 | 4.78% | 264696 | 4.80% |
|  | G to C | 6854 | 2.08% | 94216 | 1.82% | 101070 | 1.83% |
|  | C to G | 8944 | 2.72% | 124766 | 2.41% | 133710 | 2.43% |
|  | Total | 134912 | 41.00% | 2010769 | 38.79% | 2145681 | 38.92% |

**Table S4:** ﻿Summary of the goodness of fit measures.

|  | **Null.deviance** | **Df.null** | **logLik** | **AIC** | **BIC** | **Deviance** | **Df.residual** |
| --- | --- | --- | --- | --- | --- | --- | --- |
| Variation (Fig. 1e) | 6.344815225 | 131 | 814.886574 | -1619.7731 | -1605.3591 | 5.72458024 | 128 |
| Ts (Fig. 1e) | 1.987165018 | 131 | 968.83851 | -1927.677 | -1913.263 | 1.53906307 | 128 |
| Tv (Fig. 1e) | 16.54170919 | 131 | 871.423319 | -1732.8466 | -1718.4326 | 15.6183954 | 128 |
| L572 (Fig. 2c) | 24.10337753 | 131 | 223.078869 | -436.15774 | -421.74373 | 11.118054 | 128 |
| L986 (Fig. 2d) | 26.63419932 | 131 | 167.179241 | -324.35848 | -309.94447 | 14.1034737 | 128 |
| L2061. (Fig. 2e) | 18.71989888 | 131 | 247.065374 | -484.13075 | -469.71674 | 8.81765706 | 128 |
| Stop codon (Fig. 5d) | 58.90485951 | 108 | 348.06082 | -686.12164 | -672.6649 | 43.6055559 | 105 |
