# Supplementary material for: Variation around the dominant viral genome sequence contributes to viral load and outcome in patients with Ebola virus disease
Source: Genome Biol. 2020 Sep 7;21:238. doi: 10.1186/s13059-020-02148-3 (PMC7475720; doi:10.1186/s13059-020-02148-3)
Supplement: Supplementary file 2 — Additional file 2. Review history. [file 13059_2020_2148_MOESM2_ESM.docx]

Review History

**First round of review**

**Reviewer 1**

**Are you able to assess all statistics in the manuscript, including the appropriateness of statistical tests used?** Yes, and I have assessed the statistics in my report.

**Comments to author:**

Dong et al. have presented a very interesting and novel manuscript that will be of interest to researchers in the field. The study takes observations from in vivo high throughput sequencing from patients infected with Ebola virus, which come from a very unique opportunity albeit with sad consequences to many individuals. Based on these in depth sequencing data, they have formulated a hypothesis based on these observations and tested their hypothesis using functional assays in vitro. They successfully demonstrate that a virus population that has a higher frequency of minor variant mutations results in aberrant viral RNA synthesis. In vivo, they suggest that this results in a lower viral load and a better chance of survival for the patient.

The manuscript requires reworking the English (see some of my comments below). Most importantly several aspects of the analysis require clarification and additional information is required to ascertain whether biases might have been introduced.

1)     It is unclear from the analysis whether more sites show variation in the survivors versus fatalities. The sample sizes for the boxplots could be reported below the labels on the x-axis.
2)     The authors need to clarify the number of cycles used for each sample. Does the number of cycles depend on the viral load? If samples from patients with low viral load have been through a larger number of cycles, then the higher frequency of variants could be explained by the error introduced via PCR. Also, the frequency is very dependent on the coverage. Samples of low coverage may inflate the frequency of minor variants. I suggest that the authors provide a table with the statistics relating to each sample: the number of PCR cycles used, the average coverage, variation coefficient (for evenness of coverage), total number of SNPs observed in each sample and average frequency of those SNPs.
3)     Discuss whether the larger diversity observed for the survivors could be the result of a large viral population originally infecting the patient.
4)     Importantly, what is being considered as a minor variant? Are the authors carrying out any variant calling algorithms to filter low frequency variants that are the result of sequencing error? If not, was the sequencing pipeline standardised sufficiently to be able to compare the survivors versus the fatalities. As the authors are dealing with minor variants with such low frequencies, these small differences can be observed simply by having a slightly higher number of PCR cycles or running the survivors versus the fatalities on different machines.
5)     In Figure 1e, fitting different curves using a glm to the Fatalities and Survivors and testing whether there is a significant difference in the slopes and intercepts of the glm would be an appropriate test to make to show the differences between the two groups. Idem for figures 2c, 2d, 2e, figure 3e


Comments on wording and english:

I find the use of the term "average consensus sequence"  confusing as a consensus is an artificial construct based on what can be sequenced, thus not a real entity in the host. Perhaps a more appropriate term would be a master viral genome, dominant viral genome, or predominant or major viral sequence. Although, in the methodology, the use of the term consensus is appropriate.

P5L12: and supportive => as supportive
P6L1-2: "this down selected to sequence read data" => unclear
Missing "Results" heading
P9L22: mat => may
P12L11: survive infection => survive infection,
P17L10: I think the notation N572S, Q986R and F2061S is clearer.
Figure 3a: at last => at least
Figure 3 legend: compassion => comparison and repetition of "In b, d and e only the samples with at least one stop codon are shown"

**Reviewer 2**

**Are you able to assess all statistics in the manuscript, including the appropriateness of statistical tests used?** Yes, and I have assessed the statistics in my report.

**Comments to author:**

Overall, the study provides strong evidence for the reduced fitness of the LSTOP and L3mut variants of EBOV, and that these variants are more frequent in surviving acute (high Ct) patients than in fatal acute patients. The statistical methodology is sound, and the wet lab protocols are described in detail. To my knowledge, these results are novel and are consistent with previous findings on other viruses such as HepC regarding the connection between intra-host variability and clinical outcome.

My main comment is about the following conclusion from the authors: "We postulate that the minor variants in hospitalised survivors may act as both defective interfering genomes and produce aberrant or non-functional proteins, both of which may have a cumulative and deleterious effect on viral load, and push patients with EVD towards a survival outcome."

I understand that being able to reach this conclusion makes the paper much stronger in terms of its implications for anti-viral treatment. However, I think it needs further justification. As mentioned in the paper from Stumpf et al. ("Genetic diversity and models of viral evolution for the hepatitis C virus") that the authors cite with regards to the correlation between viral genome diversity and severity of clinical symptoms, the strength of the immune response of the host has an important role in the genetic diversity of the pathogen. Quoting Stumpf: "for low levels of immune response there is little selective pressure, so very few adaptive mutations will be observed, despite a large viral population size. For high levels of immune response the viral effective population size will become small and therefore selection for escape mutants will be weak."

So, my question is the following: how can we differentiate between the situation where, as the authors propose, high frequency of minor variants leads the virus population towards error catastrophe and higher host survival, versus the scenario of a stronger immune response from the host that results in increased survival as well as fewer escape mutants? Some light could be shed on this question if there were any data available on the immune response of the patients included in this study. As long as this data is not available, I think that the author's conclusion on the effect of the increased presence of minor variants would be weakened. Therefore, I think this issue should be addressed in more depth. In the current version of the manuscript, the authors do state that "both host effects and the presence of other infections will undoubtedly contribute to outcome" but they jump right away into the potential mechanistic implications of the correlation between frequency of minor variants, Ct levels, and outcome, without considering host response.

**Authors Response**

Reviewer #1: Dong et al. have presented a very interesting and novel manuscript that will be of interest to researchers in the field. The study takes observations from in vivo high throughput sequencing from patients infected with Ebola virus, which come from a very unique opportunity albeit with sad consequences to many individuals. Based on these in depth sequencing data, they have formulated a hypothesis based on these observations and tested their hypothesis using functional assays in vitro. They successfully demonstrate that a virus population that has a higher frequency of minor variant mutations results in aberrant viral RNA synthesis. In vivo, they suggest that this results in a lower viral load and a better chance of survival for the patient.

The manuscript requires reworking the English (see some of my comments below). Most importantly several aspects of the analysis require clarification and additional information is required to ascertain whether biases might have been introduced.

1)It is unclear from the analysis whether more sites show variation in the survivors versus fatalities. The sample sizes for the boxplots could be reported below the labels on the x-axis.

***We have done these analyses as requested by the reviewer. Please find the new Supplementary Fig. 2 for non-synonymous frequency variants of all sites. Please find the new Fig. 4a and Supplementary Fig. 5a. The sample sizes for the boxplots were reported below the labels on the x-axis.***

2)The authors need to clarify the number of cycles used for each sample. Does the number of cycles depend on the viral load? If samples from patients with low viral load have been through a larger number of cycles, then the higher frequency of variants could be explained by the error introduced via PCR. Also, the frequency is very dependent on the coverage. Samples of low coverage may inflate the frequency of minor variants. I suggest that the authors provide a table with the statistics relating to each sample: the number of PCR cycles used, the average coverage, variation coefficient (for evenness of coverage), total number of SNPs observed in each sample and average frequency of those SNPs.

***We have provided the additional information as requested by the reviewer. The PCR cycles were decided based on the quality and quantity of blood samples, rather than the viral load. The Supplementary Table 1 showed 86.46% fatalities and 52.63% survivors with 15 cycles. However, the survivors showed higher Tv, Ts, total variation, three protein sites (L572, L986 and L2061), and stop codon. The generalized Linear Models model (glm) showed no significant differences of slope and intercept between survivors and fatalities in any figure of Fig. 1e (and new Supplementary Table 2), suggested the survivors and fatalities have no difference of Ts, Tv and variation at same viral load (1/Ct value). Therefore, the lower viral load and read coverage could be the major reason of survivors showed a higher overall variation frequency than the fatalities. The effects of PCR cycles and read coverage on the sites should be equally across the whole genome. However, the three protein sites (L572, L986 and L2061) we reported were different from other sites. They have the lowest P-value and highest average non-synonymous frequency variants difference (>5%), separated from the major cluster (new Supplementary Fig. 2). On the other hand, these three points and stop codon in L986 showed significantly different slope and intercept between the fatalities and survivors in the glm models (new Supplementary Table 2), suggested the significant difference of non-synonymous frequency between these two groups at same viral load (1/Ct value). Therefore, the different PCR cycles would not change the results in this analysis.***

3)Discuss whether the larger diversity observed for the survivors could be the result of a large viral population originally infecting the patient.

***This is a good point for discussion, and we have substantially reworded and added to the discussion to encompass this very important point. We included examples from animal models where gain of virulence is associated with selection of minor variants. We have also considered mechanisms of how attenuating mutations could lead to persistence. We have included appropriate references.***

4)Importantly, what is being considered as a minor variant? Are the authors carrying out any variant calling algorithms to filter low frequency variants that are the result of sequencing error? If not, was the sequencing pipeline standardised sufficiently to be able to compare the survivors versus the fatalities. As the authors are dealing with minor variants with such low frequencies, these small differences can be observed simply by having a slightly higher number of PCR cycles or running the survivors versus the fatalities on different machines.

***The minor variant is any base of a site with a frequency of variation < 50%.***

***We have removed the low mapping quality and duplicated reads to improve the calling quality. We also used same method and machine for sequencing of all samples. We used the DiversiTools to call the minor variants. DiversiTools focuses on determining mutations from a population of haploid organisms, so it is working well for viruses (https://josephhughes.github.io/DiversiTools/index.html). In order to distinguish of low frequency variants from Illumina sequence errors, the DiversiTools used the calling algorithms based on the Illumina quality scores to calculate a P-value for each variant at each nucleotide site (Morelli et al. 2013) (https://bioinformatics.cvr.ac.uk/asimple-method-to-distinguish-low-frequency-variants-from-illumina-sequence-errors/). Although, DiversiTools has not been published on a peer reviewed journal, it has been used for testing frequency of mutations and cited by 17 Papers up to now (https://josephhughes.github.io/DiversiTools/index.html). We have filtered low frequency variants (P-value<0.05) and redone all analysis (almost no changes on the results).***

***Although the samples have different PCR cycles (86.46% fatalities and 52.63% survivors with 15 cycles), the effects of PCR cycles on the sites should be equally across the whole genome. However, the three protein sites (L572, L986 and L2061) we reported were different from other sites. They have the lowest P-value and highest average non-synonymous frequency variants difference (>5%), separated from the major cluster (Supplementary Fig. 2).). On the other hand, these three points and stop codon in L986 showed significantly different slope and intercept between the fatalities and survivors in the glm models (Supplementary Table 2), suggested the significant difference of non-synonymous frequency between these two groups at same viral load (1/Ct value). Therefore, the different PCR cycles would not change the results in this analysis.***

5)In Figure 1e, fitting different curves using a glm to the Fatalities and Survivors and testing whether there is a significant difference in the slopes and intercepts of the glm would be an appropriate test to make to show the differences between the two groups. Idem for figures 2c, 2d, 2e, figure 3e

***This is very good idea, many thanks. We have compared the slopes and intercepts with glm (Supplementary Table 2 and Supplementary Table 4).***

Comments on wording and english:

I find the use of the term "average consensus sequence" confusing as a consensus is an artificial construct based on what can be sequenced, thus not a real entity in the host. Perhaps a more appropriate term would be a master viral genome, dominant viral genome, or predominant or major viral sequence. Although, in the methodology, the use of the term consensus is appropriate.

***We have now changed this phraseology throughout and use ‘dominant viral genome sequence’ including in the title.***

P5L12: and supportive => as supportive

***This has been altered.***

P6L1-2: "this down selected to sequence read data" => unclear

***We have now changed this.***

Missing "Results" heading

***Now added.***

P9L22: mat => may

***Now changed***

P12L11: survive infection => survive infection,

***Sentence altered***

P17L10: I think the notation N572S, Q986R and F2061S is clearer.

***We can’t find this specifically but have tried to use throughout.***

Figure 3a: at last => at least

***This figure has been replaced but we have altered in the correct new figures.***

Figure 3 legend: compassion => comparison and repetition of "In b, d and e only the samples with at least one stop codon are shown"

***This is now changed.***

Reviewer #2: Overall, the study provides strong evidence for the reduced fitness of the LSTOP and L3mut variants of EBOV, and that these variants are more frequent in surviving acute (high Ct) patients than in fatal acute patients. The statistical methodology is sound, and the wet lab protocols are described in detail. To my knowledge, these results are novel and are consistent with previous findings on other viruses such as HepC regarding the connection between intra-host variability and clinical outcome.

My main comment is about the following conclusion from the authors: "We postulate that the minor variants in hospitalised survivors may act as both defective interfering genomes and produce aberrant or non-functional proteins, both of which may have a cumulative and deleterious effect on viral load, and push patients with EVD towards a survival outcome."

I understand that being able to reach this conclusion makes the paper much stronger in terms of its implications for anti-viral treatment. However, I think it needs further justification. As mentioned in the paper from Stumpf et al. ("Genetic diversity and models of viral evolution for the hepatitis C virus") that the authors cite with regards to the correlation between viral genome diversity and severity of clinical symptoms, the strength of the immune response of the host has an important role in the genetic diversity of the pathogen. Quoting Stumpf: "for low levels of immune response there is little selective pressure, so very few adaptive mutations will be observed, despite a large viral population size. For high levels of immune response the viral effective population size will become small and therefore selection for escape mutants will be weak."

So, my question is the following: how can we differentiate between the situation where, as the authors propose, high frequency of minor variants leads the virus population towards error catastrophe and higher host survival, versus the scenario of a stronger immune response from the host that results in increased survival as well as fewer escape mutants? Some light could be shed on this question if there were any data available on the immune response of the patients included in this study. As long as this data is not available, I think that the author's conclusion on the effect of the increased presence of minor variants would be weakened. Therefore, I think this issue should be addressed in more depth. In the current version of the manuscript, the authors do state that "both host effects and the presence of other infections will undoubtedly contribute to outcome" but they jump right away into the potential mechanistic implications of the correlation between frequency of minor variants, Ct levels, and outcome, without considering host response.

***We thank the reviewer for this major point and have tried to address this in extensively altering and refocusing the discussion. Matching data on the host cell response for many of these patients was published in Genome Biology in 2017 (Liu et al) and we have tied in the host response observed with the potential influence and implications on the dominant viral genome. We have also included a discussion of other work on the adaptive immune response and how this may alter the dominant viral genome sequence. We have noted in the manuscript in several places and also we note here that our data was based on a single snap shot of infection – that of around day 6, as represented by a diagnostic left over sample. We were not able to identify or characterise the immune response in longitudinal samples up to 14 days post infection. In many instances the European Mobile Laboratory in which the samples were gathered (which we were part) during this time was swamped and we simply did not have the capacity for such studies. We would also note, that in many fatal cases, patients were dead before the antibody response had time to act. Such studies will be difficult in the future as treatments for EVD have greatly improved.***

***The comments of the reviewer have given us time to reflect that alterations in the dominant genome that affect polymerase activity may also be a mechanism for persistence in EBOV. Normally acute infections such as EBOV are cleared (if someone survives) in two weeks. However, with EBOV, some patients get the infection again because the virus is somehow persisting. Recently, work has suggested in other similar viruses that polymerase attenuating mutations might be a potential mechanism for this. So we have included this hypothesis (and reference to it) in our work and possible interpretation of the data.***

**Second round of review**

**Reviewer 1**

I have found your improved manuscript very interesting and I think it will become a well cited article. I believe it could still benefit from some improvements in the English, I have given a non-extensive list below.

Additionally, although you have provided a bioproject number (PRJNA577693) there are currently no read files publicly available associated to this BioProject. It is important that the data is made public for future reference and reproducibility.

P41L18: The ratio of the average frequency of transversion in the
average frequency of transversion of transition => The ratio of average frequency of transversions to average frequency of transitions

P45L1: average converge => average coverage

P9L1: under same => under the same

Fig5a table: at last  => at least

P9L34: than another position => than any other position
